# Artificial Intelligence Meets Flexible Sensors: Emerging Smart Flexible Sensing Systems Driven by Machine Learning and Artificial Synapses

**DOI:** 10.1007/s40820-023-01235-x

**Published:** 2023-11-13

**Authors:** Tianming Sun, Bin Feng, Jinpeng Huo, Yu Xiao, Wengan Wang, Jin Peng, Zehua Li, Chengjie Du, Wenxian Wang, Guisheng Zou, Lei Liu

**Affiliations:** 1 Department of Mechanical Engineering, State Key Laboratory of Tribology in Advanced Equipment, Key Laboratory for Advanced Manufacturing by Materials Processing Technology, Ministry of Education of PR China, Tsinghua University, Beijing, 100084 People’s Republic of China; 2 College of Materials Science and Engineering, Shanxi Province, Taiyuan University of Technology, Taiyuan, 030024 People’s Republic of China

**Keywords:** Flexible electronics, Wearable electronics, Neuromorphic, Memristor, Deep learning

## Abstract

The recent wave of the artificial intelligence (AI) revolution has aroused unprecedented interest in the intelligentialize of human society. As an essential component that bridges the physical world and digital signals, flexible sensors are evolving from a single sensing element to a smarter system, which is capable of highly efficient acquisition, analysis, and even perception of vast, multifaceted data. While challenging from a manual perspective, the development of intelligent flexible sensing has been remarkably facilitated owing to the rapid advances of brain-inspired AI innovations from both the algorithm (machine learning) and the framework (artificial synapses) level. This review presents the recent progress of the emerging AI-driven, intelligent flexible sensing systems. The basic concept of machine learning and artificial synapses are introduced. The new enabling features induced by the fusion of AI and flexible sensing are comprehensively reviewed, which significantly advances the applications such as flexible sensory systems, soft/humanoid robotics, and human activity monitoring. As two of the most profound innovations in the twenty-first century, the deep incorporation of flexible sensing and AI technology holds tremendous potential for creating a smarter world for human beings.

## Highlights

- The latest progress of emerging smart flexible sensing systems driven by brain-inspired artificial intelligence (AI) from both the algorithm (machine learning) and the framework (artificial synapses) level is reviewed.
- New enabling features such as powerful data analysis and intelligent decision-making resulting from the fusion of AI technology with flexible sensors are discussed.
- Promising application prospects of AI-driven smart flexible sensing systems such as more intelligent monitoring for human activities, more humanoid feeling by artificial sensory organs, and more autonomous action of soft robotics are demonstrated.

## Graphical abstract

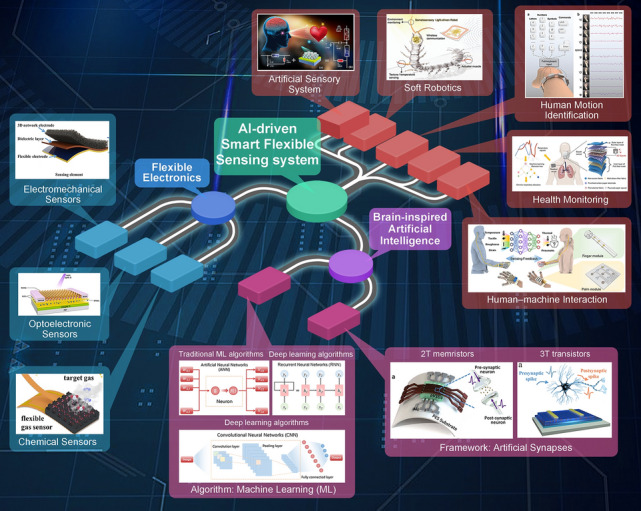

## Introduction

The continuous revolution of information technologies such as artificial intelligence (AI), big data, cloud computing, 5G/6G communications, and digital health, has prompted human life to become more interconnected and intelligent by profoundly redefining our interaction with the physical world [1–6]. Central to these transformative technologies is the field of flexible sensing, which serves to seamlessly integrate digital signals with physical spaces, which is achieved by diverse flexible sensors featured by adaptability to irregular surfaces, durability under mechanical deformation, as well as sensitivity to external stimuli [7–9]. Nowadays, the development of flexible devices is moving towards two primary trends. The first trend is the transformation from merely data acquisition to being a more intelligent system. This paradigm shift underscores the evolution of flexible sensors from single-function elements into smarter sensing systems that not only collect sensing information, but also understand and interpret the surrounding environment [10–12]. The second trend stems from the explosive growth of data volume due to the rapid development of big data and cloud computing technologies. As data become increasingly diverse and complex, there is an escalating need for the efficient processing of vast, multifaceted data [13–15]. The above two requirements are creating opportunities for machines equipped with advanced algorithms and hardware architectures, and eventually, calling for autonomous and adaptive AI that can undertake these tasks. Represented by the rapid brain-inspired advances from both the algorithm (machine learning) and the framework (artificial synapses) aspect, the ongoing wave of the AI technology revolution has made the realization of smart flexible sensing systems increasingly possible. As stated in the “Technology Roadmap for Flexible Sensors” [4], intelligence is the defining feature of the upcoming era of “Sensor 4.0”.

As one of the major subjects within AI, machine learning which focuses on the development of algorithms capable to perform tasks without explicit programming, is becoming increasingly integrated with flexible sensing [5, 16, 17]. It allows computers to learn from and make decisions based on data, thereby imitating the learning process in humans. It uses various techniques, including supervised learning, unsupervised learning, and reinforcement learning, to enable machines to improve their performance or make accurate predictions [18]. By harnessing the power of the machine learning technique, efficient post-processing, including learning from massive amounts of data, has become achievable. This technology is being preliminarily utilized in various applications such as healthcare, environmental monitoring, and human–machine interaction [19–23].

On the other hand, advanced machine learning algorithms such as Artificial Neural Networks, which originate from mimicking human brain features, are inherently incompatible with traditional von Neumann-based frameworks [24, 25]. The von Neumann framework, characterized by a separation between processing and memory units, leads to high energy consumption and limited computational power, which is the so-called “von Neumann bottleneck” [15, 26, 27]. Therefore, beyond algorithm optimizations, there is a pressing need for brain-mimicking innovations at the framework-level to fully exploit the potential of machine learning algorithms. The computational framework of the human brain offers a vastly different but remarkably efficient approach. Unlike conventional computers that process information sequentially, the human brain simultaneously processes and integrates a multitude of information streams with low power consumption [28, 29]. The essential part of the brain’s computational framework is synapses. Synapses are junctions where neurons communicate, playing a crucial role in transmitting signals and facilitating learning and memory. Each neuron can form thousands of synaptic connections with other neurons. With billions of neurons interconnected by trillions of synapses, the rapid, simultaneous processing of information is enabled. Inspired by the synaptic architecture of the human brain, researchers have developed brain-inspired synaptic devices [11, 30–32]. These devices offer a range of advantages including low power consumption, high parallelism, and real-time processing capabilities. Furthermore, some recent synaptic devices have been equipped with additional flexible features, which can be integrated with diverse flexible sensing components, contributing to the construction of intelligent sensing systems [11, 13, 33].

**Fig. 1:**
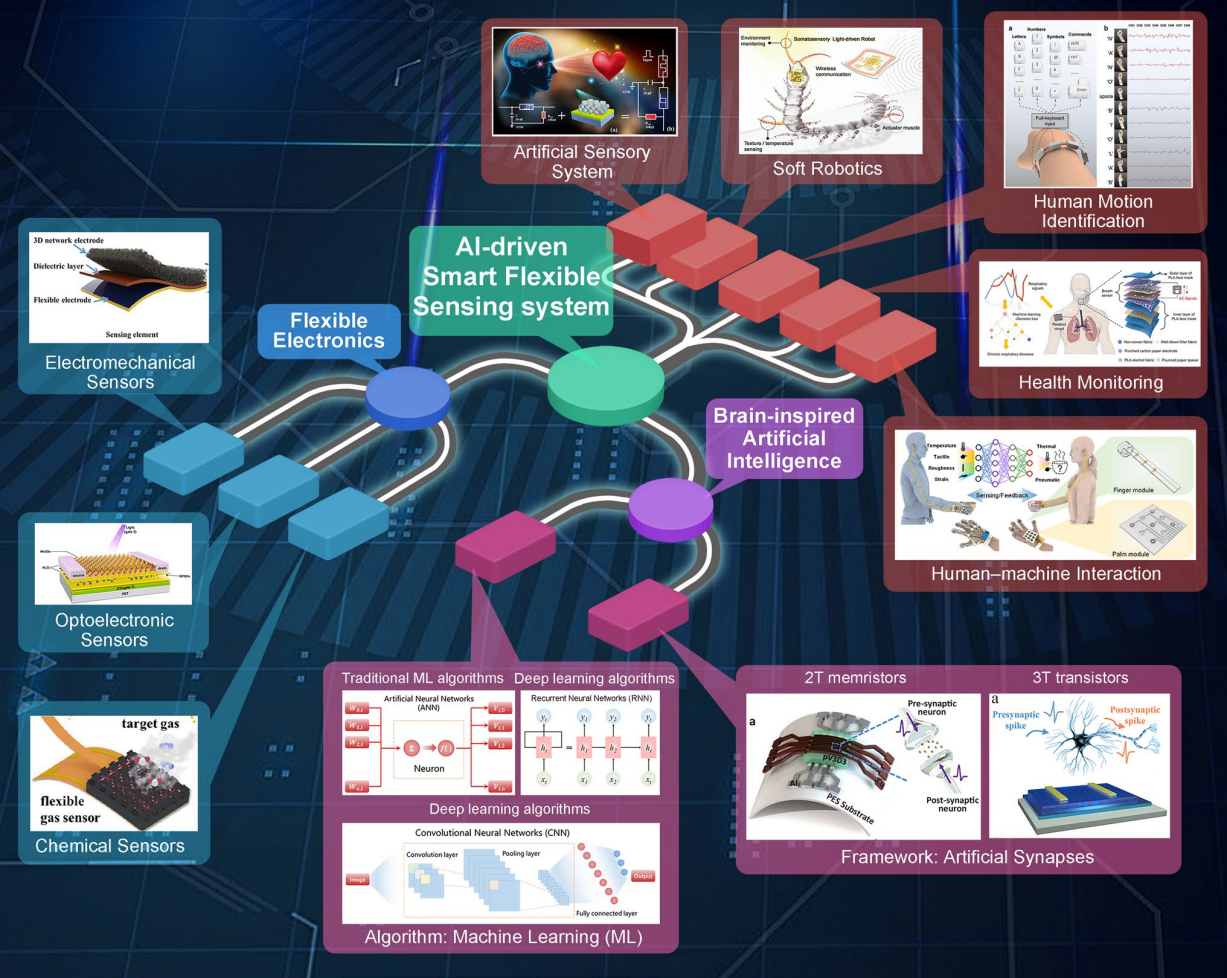
An overview of smart flexible sensing systems driven by brain-inspired AI innovations from both the algorithm (machine learning) and the framework (artificial synapses) level. Flexible electronics: electromechanical sensors [35] Copyright (2021) American Chemical Society, optoelectronic sensors [36] Copyright (2021) Elsevier, and chemical sensors [37] Copyright (2022) American Chemical Society. Brain-inspired AI: the algorithm: machine learning, and the framework: artificial synapses [38, 39] Copyright (2019) American Chemical Society, Copyright (2022) American Chemical Society. Applications based on AI-driven smart flexible sensing systems: artificial sensory systems [40] Copyright (2021) American Chemical Society, soft robotics [41] Copyright (2020) Wiley–VCH, human motion identification [42] Copyright (2022) Wiley–VCH, health monitoring [43] Copyright (2022) American Chemical Society, and human–machine interaction [44] Copyright (2022) American Chemical Society

In light of the rapid advancements of AI technology, coupled with the pressing demand for handling massive, complex data and the need for intelligence in flexible sensing, the development of AI-driven smart flexible sensor systems has emerged as a significant topic in the realm of flexible electronics. This trend underlines the transformative potential of integrating AI with flexible sensors, promising an avenue toward intelligent systems that can meet the challenges in this data-driven era (Fig. 1). This article reviews the recent progress of the development of smart flexible sensing driven by brain-inspired AI innovations from both the algorithm (machine learning) and the framework (artificial synapses) level. Although machine learning- or artificial synapses-involved flexible sensing has been separately introduced in some representative reviews [5, 11, 15, 34], we emphasize that these two AI techniques are not isolated concepts, which are comprehensively elucidated in this review. It is the incorporation of both of them that contributes to the more substantial exploitation of AI for intelligent flexible sensing. In Sect. 2, we will present the basics of the above-mentioned two AI concepts. Then in Sect. 3, we will introduce the general types of current flexible sensors, and comprehensively demonstrate the new features when flexible sensing is incorporated with machine learning and artificial synapses. In Sect. 4, we present in detail the application prospects of AI-driven flexible sensing. Finally, the key challenges and future opportunities in this emerging field are summarized and discussed.

## Basic Concepts of AI Techniques

### Principles of Machine Learning Algorithms

Machine learning is a technique that can automatically establish non-linear input–output mapping while bypassing complex physics or mathematics [45, 46]. The core idea of machine learning is to train a surrogate model using a large raw dataset, and once the model is successfully trained, its target applications such as property prediction, image recognition, and object detection can be achieved with high computational efficiency and accuracy [47]. When applying machine learning techniques in flexible sensing systems, the collected raw data should first be converted into input features for machine learning analysis by pre-processing processes such as removing outliers, denoising, and normalizations. Normally, the processed dataset is divided into three subsets, which are training, validation, and test sets [5, 18, 48]. The training dataset is utilized to train a machine learning model to approximate the input–output relationship as possible. The parameters of the machine learning model are then optimized and eventually determined by examining the validation dataset. The performance of the trained model is evaluated on the test dataset, typically with several metrics including the accuracy or confidence coefficient. Nowadays, machine learning has exhibited a remarkable significance in flexible electronics for data analysis, pattern recognition, and decision optimization, involving various fields such as medical diagnosis, health monitoring, human–machine interaction, and smart home [16, 19, 49]. Machine learning algorithms for flexible sensor systems mainly include traditional machine learning algorithms [such as support vector machines (SVM) and artificial neural network (ANN)] and deep learning (DL) algorithms [such as convolutional neural network (CNN) and recurrent neural network (RNN)] [5, 17, 34, 50].

**Fig. 2:**
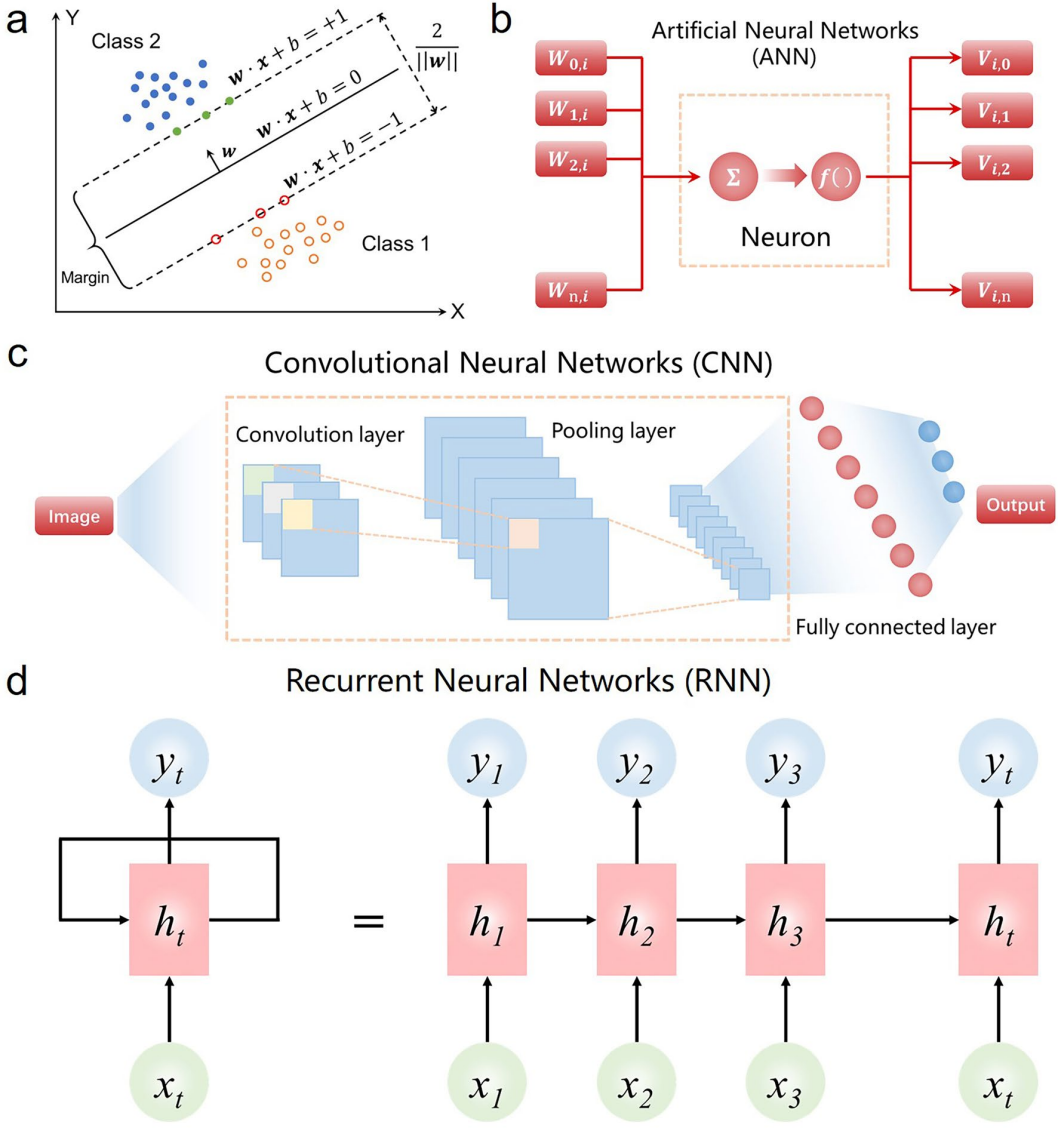
Fundamentals of various machine learning algorithms. **a** Schematic diagram of the SVM model for binary classification. **b** Architecture of the ANN model.** c** Architecture of the CNN model.** d** Architecture of the RNN model

For example, as a typical representative of traditional machine learning algorithms for classification tasks [51], SVM can separate different classes of samples by constructing an optimal hyperplane in a high-dimensional feature space (Fig. 2a). SVM can classify and identify the data sets collected by flexible electronics [52, 53]. Specifically, we can define these data points as eigenvectors. These samples are mapped from the original space to a higher dimensional feature space, making the data more easily separable in that space. Then, an optimal hyperplane is determined by some key data points (support vectors) located on the boundaries of the different categories. The new data points are mapped into the feature space and classified into a class on either side of the hyperplane. In the linear and separable training sets, the optimal hyperplane can be represented by the following equation:

1 $$w^{T} *x + b = 0$$

where *w* is the weight vector, *x* is the feature vector of the data points, and* b* is the bias term. All points satisfying Eq. (1) are on the optimal hyperplane. SVM is very effective for the classification of high-dimensional datasets and can be trained on a small dataset, but it is sensitive to noise, outliers, and the selection of parameters. In recent years, the development of machine learning based on mimicking the human brain has gradually become the research focus. For example, ANN, as a popular machine learning algorithm in flexible electronics, is a computational model that emulates the function and structure of the biological neural network (Fig. 2b) [54, 55]. Compared with other machine learning algorithms, ANN significantly outperforms the traditional machine learning model for nonlinear problems, high adaptivity, high fault tolerance, and wide applicability, and thus has better performance in several tasks, such as classification, regression, clustering, and optimization.

The DL algorithm is a new branch of the machine learning algorithm, which is significantly distinguished from the traditional machine learning algorithm. Traditional machine learning algorithms are highly dependent on feature engineering and are only applicable to relatively simple problems, while DL algorithms are capable of automatically learning feature representations and thus are suitable for handling large-scale, complex data and tasks. For the emerging DL algorithm, CNN and RNN are the two dominant structures at present.

CNN is a DL model commonly used for computer vision tasks, which consists of three main layers to accomplish the corresponding learning tasks, including the convolutional layer, pooling layer, and fully connected layer (Fig. 2c) [56, 57]. The convolutional layer is the core component of CNN, where a convolution operation is implemented on the input image by using a series of learnable filters. This convolution operation can extract local features of the image (edges, textures), which is expressed using the following equation:

2 $$\left( {I*K} \right)\left( {i, j} \right) = \mathop \sum \limits_{m} \mathop \sum \limits_{n} I(m,n)K(i - m,j - n)$$

where *I* is the input data, *K* is the convolution kernel, (I*K)(*i*, *j*) is the value of the *i* row and *j* column of the output feature map. Pooling layers can reduce the space size of the feature map by max pooling or average pooling while preserving its key features. The output of the pooling layer is flattened into a one-dimensional vector and feeds it into a fully connected neural network for the regression or classification task. In addition, compared with SVM in traditional machine learning algorithms, CNN can automatically extract features and perform operations based on image pixels. Once the training process is completed, the prediction results will be accurate and universal without the need for specialized knowledge. CNN can solve signal processing problems in flexible electronics and apply them to a variety of real-world scenarios [58].

For the RNN, its working mechanism can be simply described as a stepwise iteration through sequential data, where each moment takes the state of the current moment and the previous moment as inputs, and produces an output and a new state, which in turn becomes the input of the next moment (Fig. 2d). The state update is achieved by a recurrent unit function, which can be expressed by the formula as follows:

3 $$h_{t} = f(W_{xh} x_{t} + W_{hh} h_{t - 1} + b_{h} )$$

4 $$y_{t} = f(W_{hy} h_{t} + b_{y} )$$

where *x*_*t*_ is the input at the current moment, *h*_*t−*1_ is the state at the previous moment, *h*_*t*_ is the state at the current moment,* y*_*t*_ is the output at the current moment, *W*_*xh*_,* W*_*hh*_ and* W*_*hy*_ are the weight matrices, *b*_*h*_ and *b*_*y*_ are the bias vectors, *f*(*x*) is the activation function [59]. In practice, flexible sensing systems with multiple sensor arrays can efficiently process and recognize multi-dimensional sensing signals by connecting them to the RNN. In general, CNN is designed to automatically capture spatial features, while RNN is efficient at capturing time-series information. In addition to traditional machine learning and DL algorithms, other algorithms for data pre-processing, such as principal component analysis (PCA), linear discriminant analysis (LDA), and t-distributed stochastic neighbor embedding (t-SNE), are also involved in flexible sensor systems [34]. The typical characteristics of these commonly used machine learning algorithms in flexible electronics are systematically summarized in Table 1.

**Table 1:** Summary of the typical characteristics of various machine learning algorithms in flexible electronics [5, 17, 34, 50]

Category	Algorithms	Advantages	Disadvantages	Functions
Traditional machine learning algorithms	SVM	Very effective for the classification of high-dimensional datasets and can handle nonlinear problems	Very sensitive to noise, outliers, and parameter selection, poor interpretability of results	Classification, regression
	ANN	Strong distributed processing capabilities, high adaptivity and classification accuracy	Large data and computing resource requirements, poor interpretability, easy to overfit	Classification, regression
Deep learning algorithms	CNN	Shared convolution kernel and suitable for high-dimensional datasets, automatic feature extraction	Requires large amounts of sample data and complex parameter tuning, the pooling layer loses some information	Classification, regression
	RNN	Suitable for processing serial data, shared training parameters	Gradient vanishing and explosion, long-term dependence, low computational efficiency	Classification, regression
Data pre-processing	PCA	Great data visualization and interpretability, low computational cost of the algorithm	Lead to the loss of some detailed information, linear constraints, not applicable to non-Gaussian distributed sample data	Dimensionality reduction
	LDA	Can handle multi-category categorization and use prior knowledge of categories	Poor classification for non-linearly differentiable sample data, results in overfitting the data, large sample data requirements	Classification, dimensionality reduction
	t-SNE	Can handle high-dimensional datasets, nonlinear mapping	High randomness, high computational complexity, easy to fall into local optimal solutions	Dimensionality reduction, clustering

### Brain-Inspired Synaptic Devices

**Fig. 3:**
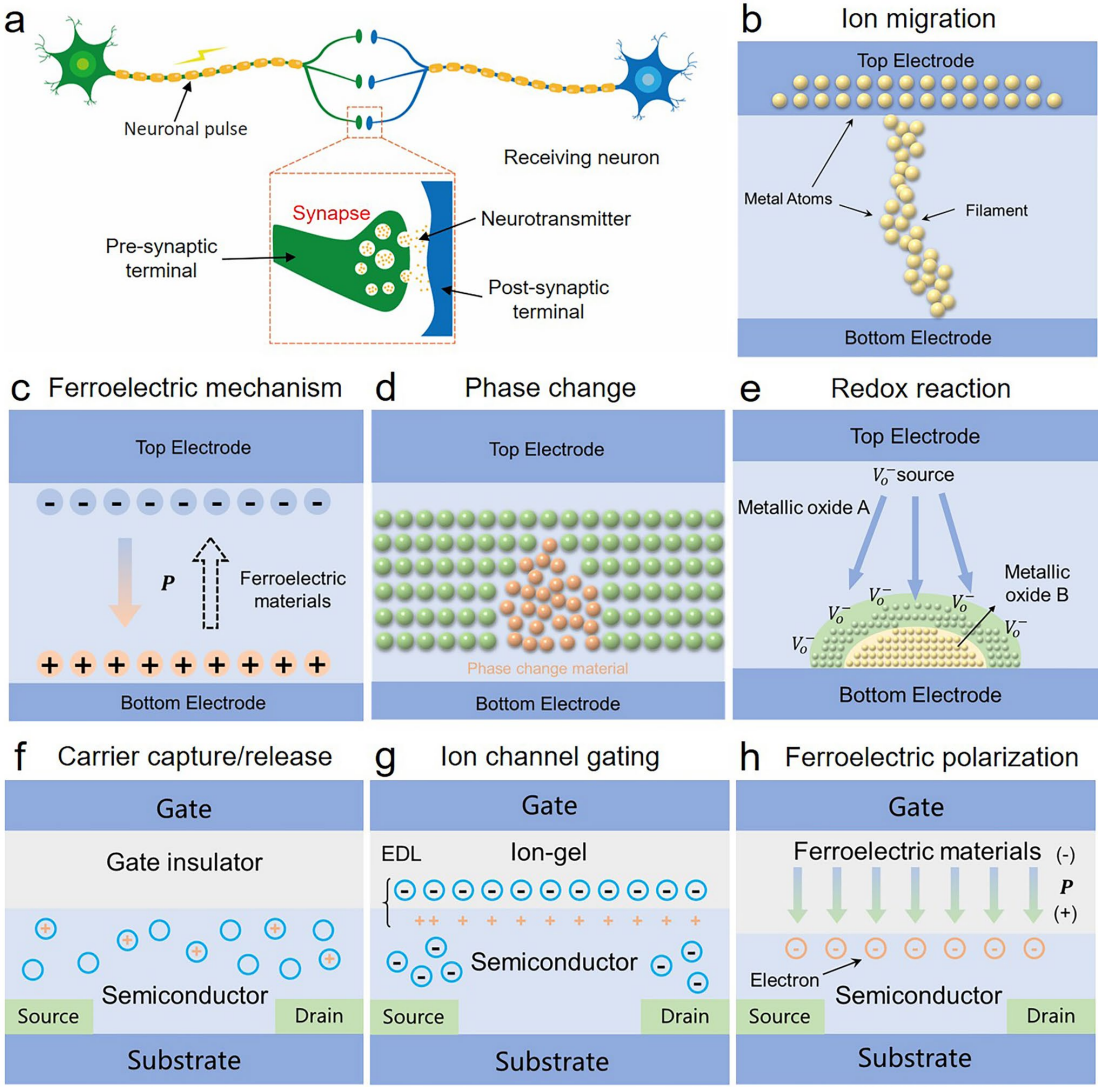
Schematic diagram of biological synapse and artificial synaptic devices based on different working mechanisms of 2 T memristors and 3 T transistors. **a** Biological synapse. **b**–**e** 2 T memristors (**b** ion migration, **c** ferroelectric mechanism, **d** phase change, **e** redox reactions). **f**–**g** 3 T transistors (**f** carrier capture/release, **g** ion channel gating, **h** ferroelectric polarization)

With the rise of AI and big data, the demand for low-energy, high-efficiency, and highly adaptive computing is gradually increasing. Emerging neuromorphic electronic systems can efficiently process massive complex information by mimicking the brain-nervous system function, which is promising to break the bottleneck of high energy consumption and slow computation of the conventional von Neumann computer architecture and facilitate the realization of brain-like intelligence [60, 61]. The biological synapse is a unique structure in the nervous system, which endows neurons to communicate and perform simultaneously computing and memory [26, 62]. To simulate this synaptic property, researchers utilize the analog and hysteresis properties of two-terminal (2 T) memristors and three-terminal (3 T) transistors to construct biomimetic synaptic components. Recently, artificial synapses based on different working mechanisms of 2 T memristors and 3 T transistors have gained great attention, as shown in Fig. 3 [11, 14].

#### Biological Neural Components

The biological synapse, as an essential structure in the nervous system, can transmit neural information, which consists of a contact point between the axon terminal of one neuron (the presynaptic terminal) and the dendritic terminal of another neuron (the postsynaptic terminal) (Fig. 3a) [63]. Several complicated synaptic behaviors are conducted by precisely controlling synaptic strength, such as information processing, learning, and memory functions [64, 65]. When a neuron is stimulated sufficiently, a nerve impulse (action potential) occurs. This nerve signal travels along the axon of neurons and reaches the axon terminal. Then, the neuron releases neurotransmitters to the synapses between neurons. Neurotransmitters can bind to receptors on the cell membrane of presynaptic neurons, leading to excitation or inhibition of the postsynaptic neuron and generating excitatory or inhibitory postsynaptic currents (EPSC/IPSC) [66]. This binding of the receptor to the neurotransmitter leads to the opening or closing of the ion channel, which alters the neuronal membrane potential and subsequently generates an electrochemical signal [67]. In addition, in the nervous system, synaptic weights (SW) refer to the connection strength between neurons, which has an important impact on the function of the nervous system by controlling the efficiency of neural signal transmission. Synaptic plasticity means the SW change in response to interactions between neurons and neural activity, which is the basis for memory and learning in the nervous system [68]. Synaptic plasticity can be divided into two categories: short-term plasticity (STP) and long-term plasticity (LTP) [69]. STP includes short-term potentiation and short-term depression, while LTP includes long-term potentiation and long-term depression. Ultimately, various types of neurons can be connected by synapses to build a complex neural network, such as the central and peripheral nervous systems [70].

#### Artificial Synapse Based on 2 T Memristors

Artificial synapse based on 2 T memristors with memory capacity and plasticity is used to simulate the synaptic connection between neurons, which comprises two conductive electrodes and an active layer of material. The synaptic response of 2 T memristors is achieved by altering the conductivity of the active material (synaptic weights). The main working mechanisms involve ion or vacancy migration, ferroelectric polarization, phase change, and redox reactions [71].

For 2 T artificial synapse devices based on ion or vacancy migration, the memristor effect is achieved by ion or vacancy migration-induced variation in the material conductivity, such as the formation and destruction of conductive filaments (Fig. 3b) [72]. Specifically, when a source voltage or electric field is applied to both ends of the electrode, the metal/halide ions or oxygen vacancies are driven by the electric field and begin to move through the material. The ion or vacancy movement can cause structural changes within the material to form conductive filaments, which increase the conductivity of the synapse device. In contrast, when the applied voltage is reversed, the ions or vacancies migrate in the opposite direction and break the conductive filament, resulting in a decrease in material conductivity. Such an effective conductivity modulation can mimic the function of a series of biological synapses, such as EPSC, IPSC, and spike-timing/rate/frequency-dependent plasticity (STDP/SRDP/SFDP) [73].

Ferroelectric materials are capable of spontaneous polarization over a range of temperatures (Fig. 3c) [74]. In 2 T memristors, ferroelectric material is commonly used as a storage layer for the storage and modulation of synaptic weights. The polarity of ferroelectric materials can be reversed under an applied voltage. In addition, the polarization state is also regulated by the electric field-induced local electrolyte reactions. Most importantly, this polarization state can be maintained for long periods of time without the need for continuous voltage or electric field drive. These processes significantly affect the material conductivity, which is used to emulate various synaptic functions, including STDP and STP/LTP [75]. In recent years, 2 T memristors based on ferroelectric materials exhibit a broad prospect in fields such as AI and neural network computing [76, 77].

Phase change materials can convert the crystalline structure from amorphous states to crystalline states in response to temperature or other external stimuli (electrical pulses and optical pulses) (Fig. 3d) [78, 79]. This structural transformation can lead to a change in the conductivity or other electrical properties of the phase change material. The memristor state can be freely switched between high resistance states and low resistance states (HRS/LRS) by varying the stimuli level. Therefore, the phase change process is used to emulate the SW variations. Nowadays, some phase change materials such as 2D materials and metal oxides are used for the construction of 2 T flexible synaptic devices, which demonstrates great potential in neuromorphic [80].

In the redox 2 T memristor, the memristor effect is realized by the formation of ion migration at the oxide/metal interface in the active material [81]. When a source voltage is imposed on the device, metal ions and oxygen ions in the material move under the electric field, thus altering the material conductivity. Once the source voltage exceeds a critical value, a redox reaction occurs between the oxide and the metal in the active material, leading to a significant modification in the chemical composition and structure at the interface (Fig. 3e). This redox reaction is usually reversible, thus 2 T memristors can be switched between different resistive states for mimicking biological synaptic function [82].

#### Artificial Synapse Based on 3 T Transistors

Compared with 2 T synapse devices, 3 T synapse devices are highly reliable and stable, which allows for multi-point input and synergistic control. In general, 3 T transistors for artificial synapses have a relatively complex structure, which is composed of three electrodes (source, drain, gate), a channel layer, and a dielectric layer. The gate electrode is responsible for regulating the electric field intensity, while the drain and source electrodes are used to control the current, similar to the role of axons, synapses, and cells in biological neurons. In a 3 T synapse device, the input signal from the presynaptic neuron is transmitted to the drain of the postsynaptic neuron by regulating the electric field intensity of the gate and controlling the magnitude of the source–drain current, thus mimicking signal transmission between the presynaptic and postsynaptic neurons [83]. The synaptic weight modification process is easily achieved by multi-gate modulation. The 3 T-based artificial synapse device can be divided into three main types according to their different working mechanisms, including carrier capture/release, ion channel gating, and ferroelectric polarization [11, 15].

In the 3 T synapse device based on the carrier capture/release mechanism, the induced carriers can migrate in the material during the stimulation by electrical or optical pulses (Fig. 3f). Some trap centers in the device can capture these carriers, which include interface defects, dangling bonds at the material surface, and barriers in semiconductor heterojunctions [84, 85]. This process can effectively regulate the conductance change of synaptic device channels. Therefore, various synaptic functions are mimicked by carrier capture/release. This mechanism is expected to enable low-energy parallel processing in the future 3 T synapse device.

Ion-gating effect mainly exploits the migration and accumulation effect of ions in the electrolyte to achieve the modulation of synaptic plasticity (Fig. 3g) [86]. Specifically, the applied gate voltage can cause ion migration in the electrolyte, resulting in a significant change in the channel layer conductivity [87]. Even in some cases, the external voltage may induce chemical reactions of oxygen ions, which further alters the electrical characteristics of the material. On this basis, 3 T synaptic devices based on the ion-gating effect are constructed for mimicking various biological synapses. Electrostatic modulation and electrochemical doping are two common ion-gating effects, which can simulate STP and LTP, respectively [88].

In 3 T synaptic devices, ferroelectric material is usually used as the dielectric layer located between the source and the drain (Fig. 3h). The external electric field can reverse the polarization direction of the ferroelectric material, which induces a redistribution of the gate charge and thus leads to a significant variation in the source–drain current [89]. Therefore, the memory behavior of the synaptic device can be realized by varying the gate voltage to regulate the source–drain current.

## AI-Driven Smart Flexible Sensing

### Common Types of Flexible Sensors

**Fig. 4:**
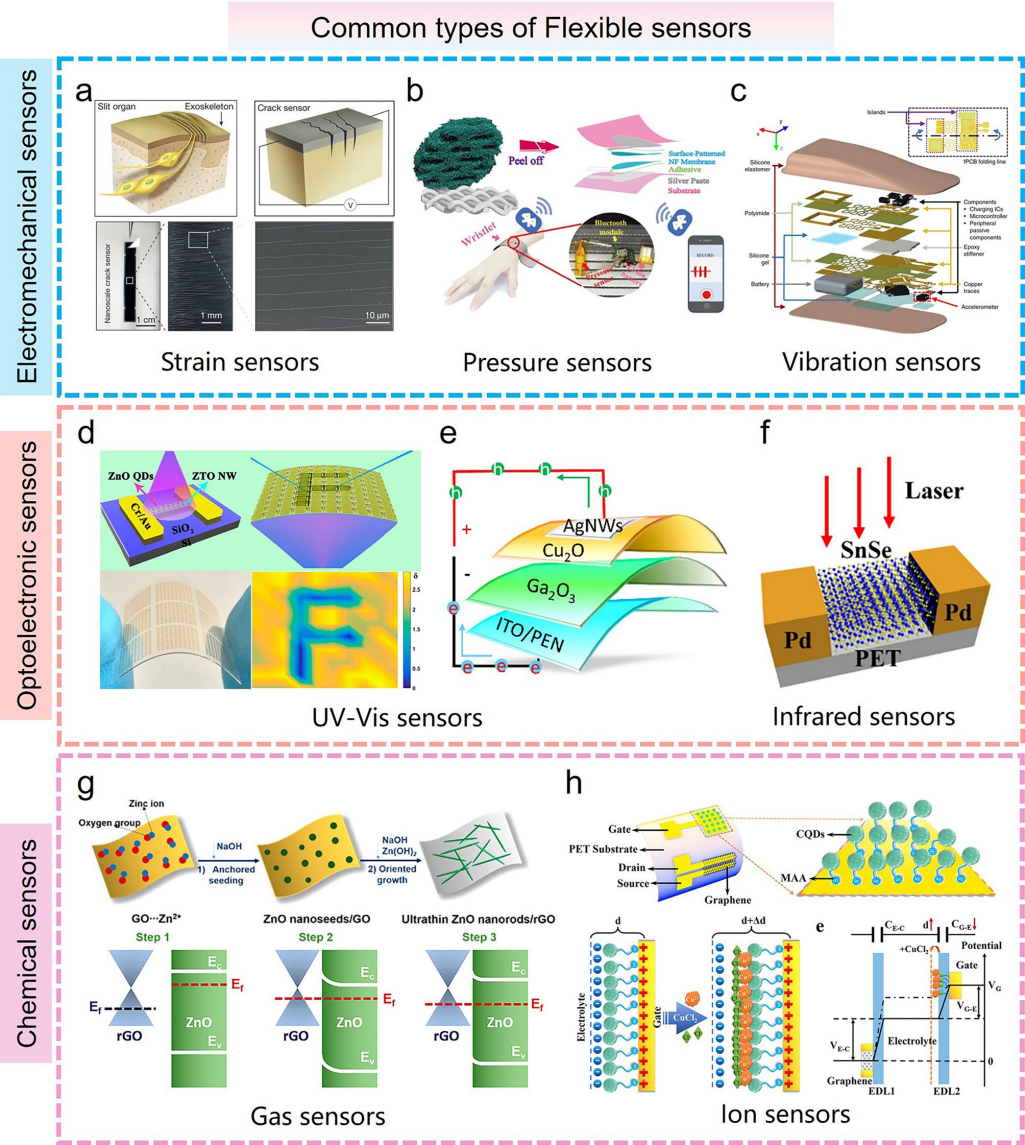
Common types of Flexible sensors. **a–c** Flexible electromechanical sensors (**a** flexible strain sensors [99] Copyright (2014) Springer Nature, **b** flexible pressure sensors [113] Copyright (2018) American Chemical Society, **c** flexible vibration sensors [126] Copyright (2019) The Authors). **d–f** Flexible optoelectronic sensors (**d, e** UV–Vis sensors [136, 137] Copyright (2017) American Chemical Society, Copyright (2022) Elsevier, **f** infrared sensors [139] Copyright (2020) American Chemical Society). **g, h** Flexible chemical sensors (**g** gas sensors [153] Copyright (2016) American Chemical Society, **h** ion sensors [155] Copyright (2020) American Chemical Society)

Nowadays, flexible electronics are essential for human beings to pursue intelligent life [90, 91]. Various complex, dynamic, non-planar service scenarios, such as healthcare, human–computer interaction, signal monitoring, and soft robotics, urgently need the participation of wearable devices to obtain high-quality data information [92]. The booming flexible sensors are divided into three main categories according to the type and function, including flexible electromechanical sensors, flexible optoelectronic sensors, and flexible chemical sensors (Fig. 4), which are building blocks for the further development of various intelligent sensing systems introduced in the following sections.

#### Flexible Electromechanical Sensors

Flexible electromechanical sensors transduce mechanical stimuli into electrical signals to collect information about the corresponding objective. These mechanical stimuli involve various pressures, strains, vibrations, and shear stresses. For instance, flexible strain sensors can detect mechanical stress or strain by measuring the material deformation, which is usually composed of flexible sensing materials and substrates, thus allowing them to bend and adapt to various surface shapes. These features enable flexible strain sensors to be used in a variety of complex and dynamic environments, such as human skin, wearable devices, and robots [93–95]. Various sensing materials for flexible strain sensors include metal nanoparticles/nanowires/film, metal oxide, carbon micro/nanomaterial, liquid metal, conductive polymer, and transition metal dichalcogenides [96–98]. When the sensor is subjected to an external mechanical deformation, the electrical characteristics of the sensing material will vary in real time and generate the corresponding electrical signal output. For the strain sensor with high sensitivity, it usually presents a high gauge factor (GF > 5000), low detection limit (strain < 1%), and fast response time. Therefore, such a type of strain sensor is very suitable for detecting small strain, including sound vibrations, subtle physiological signals, and engineering applications. For example, several common physiological signals of pulse and heart rate have been successfully captured by Choi's group and Liu's group utilize this highly sensitive sensor (Fig. 4a) [99, 100]. Moreover, the speech recognition function for mute expression and pronunciation calibration is achieved by capturing the vibration of the human throat during speaking [101–103]. For the strain sensor with a relatively large working range, a representative application is motion monitoring of human joints, including swallowing, blinking, finger bending, wrist bending, elbow bending, and knee bending, which is extremely important for obtaining human physiological details [104–107]. Based on the monitoring of this information, several interesting functions such as information coding [100], sign gesture translation [108, 109], and braille recognition [6] can be successfully implemented to help disabled people.

In addition to flexible strain sensors, flexible pressure sensors are also a common electromechanical sensor, which can accurately measure pressure variations on the object’s surface. Over the years, several flexible pressure sensors have been systematically investigated for mimicking the tactile sensing function of human skin, as illustrated in Fig. 4b [110, 111]. For instance, flexible piezoresistive sensors include an active material sandwiched between two opposing flexible electrodes. The active material generally comprises a composite of a conductive material and an elastic matrix to form a porous structure, whose resistance can be expressed by the resistivity of the material (ρ), the length (L), and the cross-sectional area (A) as: *R* = *ρL*/*A*. Pressure-induced deformation significantly reduces the resistance of the active material by increasing the contact area of the conductive material. To date, several common active materials have been used for flexible piezoresistive sensors, including conductive materials [metal NPs/NWs (Au, Ag, Pt, Cu), 2D materials (MXene, graphene, MoS_2_)] [112, 113] and elastic matrix (PDMS, Ecoflex, and textiles) [114–116]. Flexible piezoresistive sensors with lightweight, high accuracy and ease of integration are widely used in healthcare, motion monitoring, and robotics. Compared with flexible piezoresistive sensors, flexible piezoelectric sensors can respond to external pressure by generating a transient electrical signal, which consists of a piezoelectric material sandwiched by two parallel flexible electrodes. When the sensor is subjected to external pressure, the deformation of the piezoelectric material can cause electric polarization, inducing charge distribution and spatial potential difference. Currently, several common piezoelectric materials have been developed, such as piezoelectric polymers (PVDF) [117, 118], lead zirconium titanate (PZT) [119, 120], gallium nitride (GaN) [121], and zinc oxide (ZnO) [122]. Flexible piezoelectric sensors with excellent dynamic response, high sensitivity, and resolution are suitable for the detection of dynamic pressure and high-frequency vibrations.

Similar to pressure sensors, flexible vibration sensors that are sensitive to vibration are primarily used to measure the vibration and dynamic strain of machines or structural objects. This sensor typically consists of a flexible substrate and a sensing element. The sensing element works on the principles of capacitance, resistance, piezoelectricity, or resonance. In nature, collecting vibration signals with different frequencies and amplitudes are extremely important for medical diagnosis, motion measuring, and device detection [123, 124]. As a demonstration, flexible vibration sensors have been used to monitor human movement and physiological signals such as heart rate, respiration, pulse, and posture (Fig. 4c) [125, 126]. These vibration signals have a relatively low frequency (0–100 Hz). For the ultra-high frequency range (kHz–MHz), the flexible vibration sensor is commonly used for ultrasonic-based structural health monitoring [127, 128].

#### Flexible Optoelectronic Sensors

Over the past decade, the booming flexible optoelectronic sensors have attracted the attention of many researchers, which transduce light energy into electrical signals and detect optical information of objects [129, 130]. Flexible optoelectronic sensors with lightweight, shape deformability and ease of integration are widely applied in various optical detection, visual perception, and energy field. Currently, according to the different detection ranges, flexible optoelectronic sensors mainly include ultraviolet–visible (UV–Vis) sensors and infrared sensors (Fig. 4d–f).

UV–Vis sensors, as one of the most common optoelectronic sensors, are mainly suitable for detecting light signals in the ultraviolet and visible wavelength ranges [131, 132]. For instance, Kim’s group developed an ultrasensitive photodetector with a high photodetectivity (6 × 10^10^ Jones) and photoresponsivity (1.031 A W^−1^) to detect light at wavelengths from 350 to 700 nm by utilizing multilayer MoS_2_ as the channel material [133]. Similarly, Nawaz et al*.* [134] reported for the first time a hybrid 1D CdSe nanoribbon/2D PbI_2_ nanosheet heterojunction device for high-performance flexible photodetectors. This device is sensitive to UV–Vis light (200–800 nm) and characterized by high responsivity (3.98 × 10^6^ A W^−1^), good detectivity (8.62 × 10^16^ Jones), large linear dynamic range (76 dB), and external quantum efficiency (9.83 × 10^8^%). Wu et al*.* also demonstrated a flexible perovskite (MAPbI_3_) nanowire network (NWN) photodetector based on a simple welding strategy for responding to ultraviolet and visible light. This NWN-based photodetector exhibits ultra-high photoelectric performance due to weld-enhanced material crystallinity, including a high on/off ratio (2.8 × 10^4^) and detectivity (4.16 × 10^12^ Jones) [135]. Featuring these excellent performances, such a kind of sensor exhibits a broad prospect in the fields of smart wearable devices, biomedical imaging, environmental energy, and security. Shen et al*.* have fabricated a high-performance flexible ultraviolet image sensor with a 10 × 10 device pixel array, which is extremely beneficial for the development of large-area flexible sensing systems (Fig. 4d) [136]. Moreover, several high-performance UV–Vis sensors have been developed as flexible photovoltaic devices, which play a key factor in the production of sustainable and environmentally friendly energy. Kumar et al*.* reported a flexible, lightweight, and transparent Ga_2_O_3_/Cu_2_O heterojunction for UV–Vis photodetectors and photovoltaics, as shown in Fig. 4e. This device with specific UV shielding function and energy production capabilities can be easily integrated into various medical devices and smart wearable systems and provide energy supply [137]. Notably, the integration of these photodetectors and storage devices even allows for the development of advanced flexible visual memory systems to emulate the human visual memory function [138].

In contrast to UV–Vis sensors, infrared sensors are commonly used to detect light signals in the infrared wavelength range. Infrared light is typically between visible light and microwaves, with wavelengths ranging from 750 to 1 mm. It can be further divided into near-infrared light (750–3 μm), mid-infrared light (3–30 μm), and far-infrared light (> 30 μm). Great efforts have been made to develop various high-performance infrared sensors with ultra-broad spectral responses. Xu et al*.* [139] designed a novel flexible SnSe-based photodetector, which can even detect ultraviolet–visible-mid-infrared light (10.6 μm) by photobolometric effect and exhibit ultra-high mechanical stability and fast response rate (Fig. 4f). Furthermore, an In_2_O_3_/PTPBT-ET-based hybrid phototransistor has been developed by Li's group in 2021, which demonstrates high performance in near-infrared light sensing, such as high responsivity (200 A W^−1^), excellent detectivity (1.2 × 10^13^ Jones), fast rise/fall time (5/120 ms) and outstanding mechanical durability (1000 bending cycles). This device is further used for flexible near-infrared image sensors by constructing 10 × 10 phototransistor arrays [140]. Nowadays, infrared sensors have been widely used in the fields of night vision, thermal imagery, security monitoring, and medical analysis [141, 142]. In addition to UV–Vis sensors and infrared sensors, there is a special terahertz (THz) sensor with a frequency range of 0.1–10 THz and a wavelength of > 30 μm. This THz sensing technology is extremely important for medical imaging, non-destructive testing, and 5G/6G communications [143].

#### Flexible Chemical Sensors

In contrast to the previous two flexible sensors, flexible chemical sensors that are sensitive to various chemicals can convert their chemical information into read-out electrical signals for detection [144–146]. These sensors are similar to the olfactory and gustatory organs in the human sensory system. Compared with the traditional fluorescent and labeled chemical sensing technology, the emerging flexible chemical sensors with high sensitivity and specificity and low detection limit exhibit broad prospects in the fields of chemical environmental pollution, sanitary surveillance, and medical health, including the detection of various gas molecules, organic solvents, volatile organic compounds (VOC), ion, bio-enzyme, and virus molecule [147–149]. In general, the operating mode of the sensor is closely associated with its functional sensing material. For flexible gas sensors, the fundamental principle is a chemical reaction between the gas sensing material and the adsorbed gas molecular, which induces a significant variation in the electrical properties of the material. Therefore, the composition and concentration of the gas can be effectively detected based on the output electrical signal. Therefore, the composition and concentration of the gas can be effectively detected based on the output electrical signal. Several common oxide semiconductor materials, such as SnO_2_, ZnO, and Fe_2_O_3_ are widely used to fabricate the flexible gas sensor [150, 151]. Yang et al*.* [152] have demonstrated a transfer-free graphene growth method to achieve a fast and simultaneous response to nitrogen dioxide (NO_2_) and toxic ammonia (NH_3_) molecules. Similar works to detect the NO_2_ gas were completed by a-Fe_2_O_3_ and ZnO-modified reduced graphene oxide nanocomposites (Fig. 4g) [153, 154]. The detection of these pollution gases is essential for environmental protection and ecological safety. In addition, ion sensors are also common chemical sensors that detect and quantify the presence and concentration of various ions by the interaction between sensing materials and specific ions. Over the past decade, various ion sensors have made significant progress in environmental monitoring, intelligent medicine, and food safety. These sensors can be used to detect heavy metal ions and drug residues in water, or ion levels in human fluids. For example, Fan et al*.* [155] reported a solution-gated graphene transistor sensor platform to selectively detect Cu^2+^ ion in the various interference metal ions based on the affinity between the functional amine group of carbon quantum dot and the Cu^2+^ ion (Fig. 4h). Dahiya’s group developed a stretchable wireless system for the pH monitoring of human sweat [156].

In addition to gas sensors and ion sensors, there are several special chemical sensors, such as biological sensors, VOC sensors, humidity sensors, sweat sensors, blood oxygen sensors, and glucose sensors [157, 158]. Biological sensors can monitor physiological or chemical indicators inside or outside the organism. These detection targets include nucleic acids, proteins, pathogens, cells, and various small biomolecules (lactate, urea, dopamine, and H_2_O_2_), which are closely associated with human health [159, 160]. VOC sensors are mainly used for the monitoring of organic compounds with volatile, toxic, and harmful characteristics, which are directly relevant to human health [161] and ecological safety [162]. Humidity sensors are commonly used to measure the water vapor content in the air or other gases, which is essential for air quality monitoring. The sweat sensor is specifically designed to detect the composition and secretion rate of human sweat, while the role of the blood oxygen sensor and glucose sensor is to monitor human blood oxygen and blood glucose levels. These chemical sensors are expected to build future artificial olfactory and gustatory perception systems.

### Machine Learning-Driven Smart Flexible Sensors

With the increase in the number of flexible sensors and the amount of data, the processing and analysis of sensing information become more and more complex and difficult. Due to the advantages of processing large-scale data, adaptive capability, high efficiency, automation, and intelligence, the incorporation of machine learning has had a profound impact on the field of flexible electronics in recent years, making it more intelligent and connected by adding a powerful tool for analyzing and processing data from multiple types of sensors [163]. Specifically, machine learning plays a key role in three main aspects: sensing data analysis and interpretation, multimodal information post-processing and decoupling, and intelligent environment sensing and perception (Table 2).

**Table 2:** Recent progress in machine learning-driven smart flexible sensors to enable sensing data analysis, multimodal information processing and decoupling, and intelligent environment perception

Category	Sensor type	Machine learning algorithms	Application	References
Sensing data analysis and interpretation	Triboelectric sensors	SML	Ambulatory cardiovascular monitoring	[164]
	Self-powered breath sensor	DT	Chronic respiratory disease diagnosis	[43]
	Triboelectric pressure sensors	DL, CNN	Gait analysis, VR applications	[165]
	Piezoelectric acoustic sensors	GMM, DNN, CNN, t-SNE	Voice communication, speaker recognition	[49]
Multimodal information processing and decoupling	Skin-like stretchable strain sensors	CNN, t-SNE, BSV	Gesture recognition	[166]
	Piezoelectric tactile sensors	DL, CNN	Tactile Cognition	[167]
	Transient multifunctional sensor	KNN, DT, RF, ET	Non-invasive personal care diagnostics	[168]
	Hybrid electronic system	CNN	Ambulatory physiological monitoring	[169]
	Epidermal electronic sensors	SVM, KNN, DT	Mental fatigue monitoring	[170]
Intelligent environment sensing and perception	Triboelectric nanogenerator sensors	PCA, SVM	Digital twin applications	[171]
	Triboelectric sensors	CNN, PCA, DL	Sign language recognition and bidirectional communication	[172]
	Piezoresistive sensors	CNN	Object recognition and grasping	[173]
	Piezoresistive sensors	ANN, SVM	Sitting posture recognition	[178]
	Triboelectric sensors and image sensor	CNN, t-SNE	Integrated health monitoring system, smart home	[175]

*SML* supervised machine learning, *BSV* bioinspired somatosensory-visual, *RF* random forests, *ET* extra trees

**Fig. 5:**
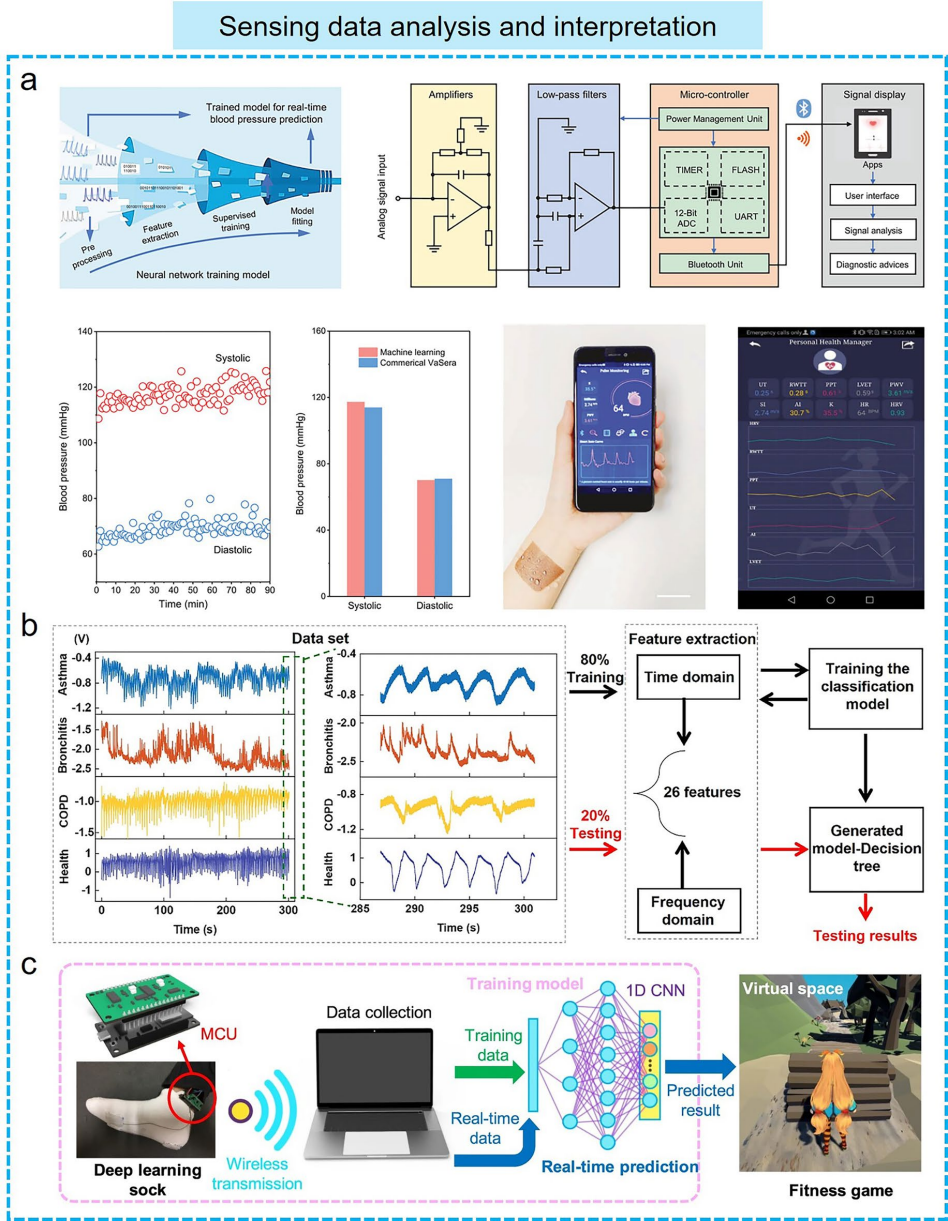
Machine learning algorithms enable sensing data analysis and interpretation in different flexible electronic applications. **a** Ambulatory cardiovascular monitoring system [164] Copyright (2021) Wiley–VCH. **b** Chronic respiratory disease diagnosis [43] Copyright (2022) American Chemical Society. **c** Gait analysis [165] Copyright (2020) The Authors

First, machine learning algorithms can help flexible electronics efficiently process massive amounts of sensor information (Fig. 5). Currently, the data collected by flexible sensors are becoming larger and more complex, requiring processing, filtering, and extraction. Traditional rule-based methods face difficulties in handling such large-scale data, while machine learning algorithms can process and analyze the data quickly and efficiently by training models to learn features and patterns from the data, thus enabling flexible electronics to make intelligent data decisions. Several machine learning algorithms have been successfully used to analyze the physiological signals collected by flexible electromechanical sensors for health monitoring to further understand human health status. Fang et al*.* [164] designed a machine learning-assisted textile triboelectric sensor to achieve ambulatory cardiovascular condition assessment and high-fidelity pulse monitoring (Fig. 5a). In this system, machine learning algorithms are used to extract pulse wave features from the data collected by the triboelectric sensor, enabling cuffless blood pressure estimation. This information is subsequently used as inputs to the trained neural network, which generates two outputs corresponding to systolic and diastolic blood pressures. This machine learning-assisted estimation of blood pressure is quite reliable due to the relatively small mean deviation from commercial cuff-validated values (2.9% and 1.2%, respectively). Besides pulse wave feature extraction, machine learning algorithms are used for the diagnosis of some medical diseases diagnose, such as respiratory disease classification and benign/malignant tumor identification. An air-permeable and biodegradable smart face mask has been successfully developed, which is composed of a self-powered breath sensor, a mobile readout circuit, and a polylactic acid-made mask [43]. This smart mask enables the effective diagnosis of chronic respiratory diseases by recording respiratory signals and combining them with machine learning algorithms (Fig. 5b). Specifically, each complete respiratory waveform is considered as a dataset, which includes 26 typical features based on the frequency and time domains. On this basis, a total of 2400 datasets were obtained, of which 80% were used as training sets for training the model and 20% were used as test sets to complete the validation task. As a typical example, this smart mask, assisted by the decision tree (DT) algorithm based on a bagged ensemble strategy, has successfully achieved the differentiation between the healthy group and three typical chronic respiratory diseases (chronic obstructive pulmonary disease, asthma, and bronchitis) with an overall accuracy of 95.5%. In addition to health monitoring, the efficient data processing and analysis capabilities enabled by machine learning algorithms are also used in several human–machine interface situations, such as voice communication and speaker recognition, which are commonly integrated into a variety of flexible acoustic pressure sensors to enhance their functionality. An interface platform for speech users was developed by combining flexible piezoelectric acoustic sensors and machine learning techniques [49]. In this platform, the acoustic sensor vibrates in response to the speaker's voice and subsequently transforms speech into an electrical signal, which can supply digital data for pre-processing. Machine learning models are used to train these speech data and extract linguistic information from it, which is essential for the development of voice-activated electronic systems. Furthermore, machine learning algorithms can also be combined with several flexible electromechanical sensors to achieve accurate identification and evaluation of human movements and postures by real-time data processing and analysis, providing support for personalized motion monitoring and guidance, and enabling real-time feedback. Lee’s group developed an intelligent sensing system for sophisticated gait analysis by connecting low-cost triboelectric intelligent socks with an optimized DL model (Fig. 5c) [165]. The smart sock equipped with 1D CNN-based DL analysis was capable of distinguishing gait patterns with 100% and 93.64% accuracy in 5 and 13 participants, respectively. It was also able to detect various human activities with an accuracy of 96.67% among the five predefined actions of the identified users. In short, this machine learning-based data analysis capability can improve the perception and intelligence of flexible electronics, which is significant to better cope with complex environments and task requirements.

**Fig. 6:**
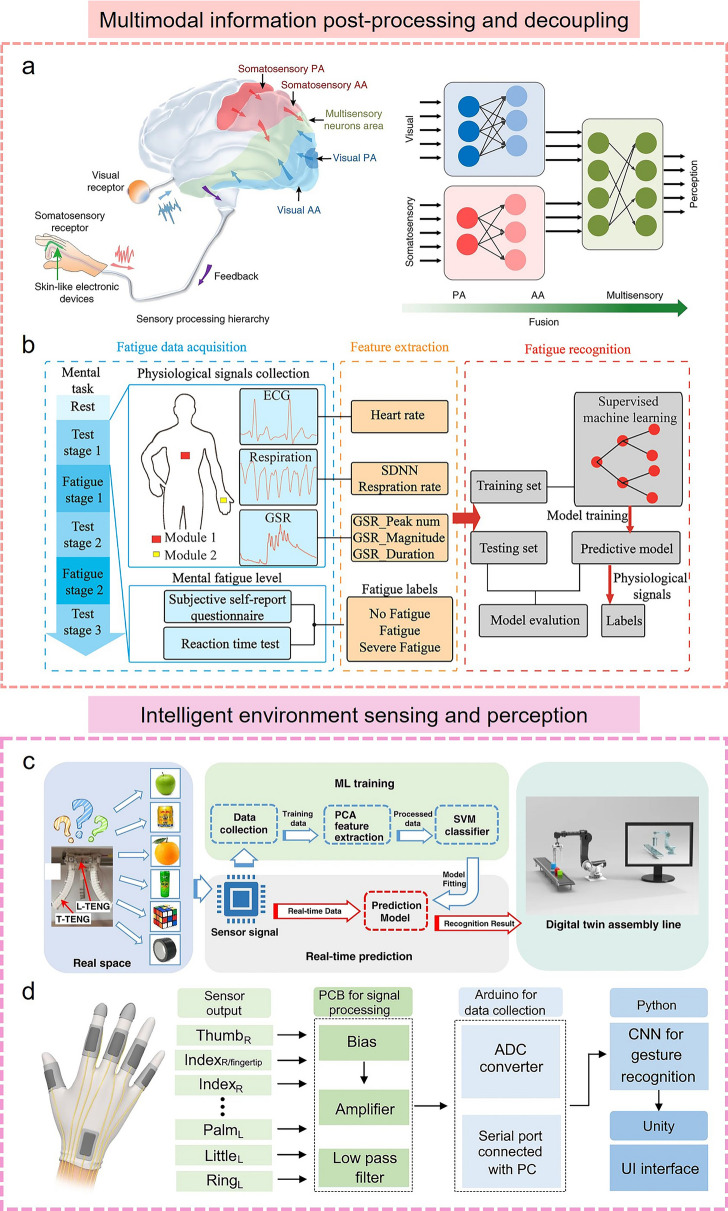
Machine learning algorithms help flexible electronics to achieve **a, b** multimodal information post-processing and decoupling, **c, d** intelligent environmental sensing and perception in different scenarios. **a** Fusion of visual and somatosensory information in the BSV-associated learning architecture [166] Copyright (2020) The Authors. **b** Mental fatigue status monitoring system with machine learning algorithms [170] Copyright (2020) American Chemical Society. **c** Digital twin unmanned warehouse system with machine learning algorithms [171] Copyright (2020) The Authors. **d** AI-enabled sign language recognition and communication system [172] Copyright (2021) The Authors

Moreover, machine learning algorithms can help flexible electronics to perform complex data fusion and multimodal information processing. Some flexible sensors generally integrate multiple types of sensors to acquire multi-dimensional information, such as vision, sound, and pressure. Machine learning algorithms can fuse or decouple these various types of sensor data to extract useful features and patterns for the processing and understanding of complex multimodal information, which enables flexible electronics to perform more comprehensive and accurate data analysis and decision-making in different fields such as human–machine interaction, health monitoring, and smart home. For instance, a bioinspired data fusion architecture was developed by Chen's group for human gesture recognition [166]. This process is implemented by combining somatosensory data with visual data. The bioinspired learning architecture performs vision processing utilizing a sectional CNN, and then enables sparse neural networks for feature-level sensor data fusion and recognition, thus achieving a high recognition accuracy even under non-ideal conditions of noisy, underexposed or overexposed images (Fig. 6a). Similarly, Kim et al*.* [167] reported a tactile avatar system for mimicking human tactile cognition by integrating a multiarray piezoelectric tactile sensor and a DL process. In this system, piezoelectric tactile sensors are responsible for dynamically recording different tactile information, such as temperature, pressure, hardness, sliding speed, and surface topography. Using a hybrid neural network layer, the haptic decision system is realized by training multimodal tactile sensory information collected by touch or swipe and creating individualized histograms of human tactile cognition. CNN algorithms were used to generate artificial tactile perceptions in 42 different tactile materials with less than 2% decision error in each avatar system. In addition, machine learning algorithms can help identify various stimuli from the signals of complex multifunctional sensors. Sahatiya et al*.* demonstrated a multifunctional sensor for detecting physical and chemical stimuli utilizing a water-soluble SnS_2_ QD/PVA film and further combined with machine learning algorithms to achieve accurate classification of various sensor data [168]. These multifunctional sensor data were processed by various machine learning algorithms (KNN, DT, random forests, extra trees) in which the data were trained to decouple and classify strain, pressure, and respiratory stimuli with a maximum accuracy of 87.7%. Recently, researchers have also used machine learning algorithms to process and analyze various physiological signals and decouple and classify this raw data in real time. A wireless, stretchable flexible electronic system has been developed to monitor the user's heart condition in real time by continuously evaluating, detecting, and notifying the recorded ECG [169]. In this system, two CNN units were used to classify user activities such as idling, walking, stairs, running, and falling based on acceleration and angular velocity data, as well as to perform semantic segmentation of ECG ectopic beats (ventricular ectopic beats, supraventricular ectopic beats, and fusion beat) and arrhythmias (myocardial infarction and heart failure) for heart disease based on raw ECG data. Furthermore, a recent advancement in the field saw the development of multimodal epidermal electronic systems, designed to monitor multiple physiological signals, including ECG, GSR signals, and respiration rate in a nonintrusive way [170]. By introducing machine learning algorithms to extract the key features of the corresponding physiological signals, a mental fatigue classification system is developed to enable high-precision prediction of fatigue levels (Fig. 6b). SVM, KNN, and DT algorithms were used to complete the model training and build the prediction model. By utilizing the DT algorithm, a prediction rate of 89% was realized based on six different physiological characteristics. The complex multimodal information fusion and decoupling capabilities endowed by the machine learning algorithm bring many advantages to flexible electronics, not just simple data processing and decision support, but more of an increased level of intelligence.

More importantly, the combination of machine learning-assisted data processing and flexible electronics can endow some inanimate objects such as soft robots, smart gloves, and prosthetics with the ability to perceive information and interact with the environment, enabling them to better understand and respond to user demand, thus creating more personalized services and more intelligent interaction experience for human users. Recently, a smart soft-robotic gripper system has been designed to capture continuous motion and tactile information [171]. In this smart system, tactile sensors can sense the contact location and area of external stimuli through specially distributed electrodes. Subsequently, the tactile sensory information collected by operating the soft gripper was further trained by an SVM algorithm to recognize different objects with an accuracy of 98.1%, and the digital twin application demonstration based on the virtual assembly line and unmanned warehouse applications was successfully created (Fig. 6c). In addition, several intelligent perception systems integrated with machine learning algorithms and wearable sensors have been developed to achieve comprehensive gesture recognition, thus meeting the daily communication needs of sign language users. Lee et al*.* reported an AI system for sign language recognition and communication, which is composed of smart sensing gloves integrated with triboelectric sensors, DL block, and a virtual reality interaction interface (Fig. 6d) [172]. As proof, the system successfully achieved the recognition of 20 short phrases and 50 words. In this system, CNN models were used to enhance the algorithm's ability to recognize objects, while DL algorithms are used to identify linguistic components and reverse the construction of the original phrase, thereby providing an accurate translation function by realigning known word units to generate new sentences. Finally, a virtual reality interface was designed to facilitate communication between disabled people based on the DL-assisted glove smart system. Similar work was demonstrated by Matusik’s group, who designed a tactile glove with an array sensor and used a CNN to analyze the array data to recognize individual objects, evaluate the weight, and investigate the classic tactile patterns that occur when grasping objects [173]. Currently, the intelligent sensing and decision-making capabilities of wearable electronics empowered by machine learning algorithms also show broad prospects in the smart home. Hu et al*.* [174] reported an intelligent chair sitting recognition system that uses six flexible piezoresistive sensors, a machine learning algorithm of the two-layer ANN, and an analog-to-digital converter (ADC) board to classify seven different health-related sitting postures. Moreover, an artificially intelligent toilet integrated with image sensors and pressure sensor arrays was developed for an integrated health monitoring system [175]. Assisted by CNN algorithms, the smart toilet can recognize the user information sitting on the toilet seat with over 90% accuracy.

In a word, different machine learning algorithms have distinct characteristics, which mainly include data pre-processing algorithms and analysis algorithms. In these smart flexible sensing systems, the flexible sensor module first acquires massive raw data containing various stimuli information. These raw data, differentiated from the data format required for machine learning, are pre-processed or transformed to extract feature information such as time, frequency, amplitude, and polarity. PCA and LDA are commonly used data pre-processing algorithms that enable dimensionality reduction of high-dimensional datasets in flexible electronics. Similar functions can also be achieved by the t-SNE algorithm, but it is only suitable for the nonlinear case. These processed data are further used for model training. To date, some machine learning models are used to perform different training tasks. For example, several traditional machine learning algorithms (SVM, ANN) and deep learning algorithms (CNN, RNN) can implement classification and regression tasks. Other algorithms such as k-means and Gaussian Mixture Model (GMM) can accomplish the clustering task. The target of these training tasks is to enable machine learning models to make analyses and predictions about the collected information, and thus achieve a smart flexible sensing system with self-learning and decision-making capabilities. In the future, machine learning-driven smart flexible sensors will certainly inspire more sophisticated and intelligent wearable product design, providing convenient and comfortable services for humans.

### Artificial Synapse-Driven Smart Flexible Sensors

**Fig. 7:**
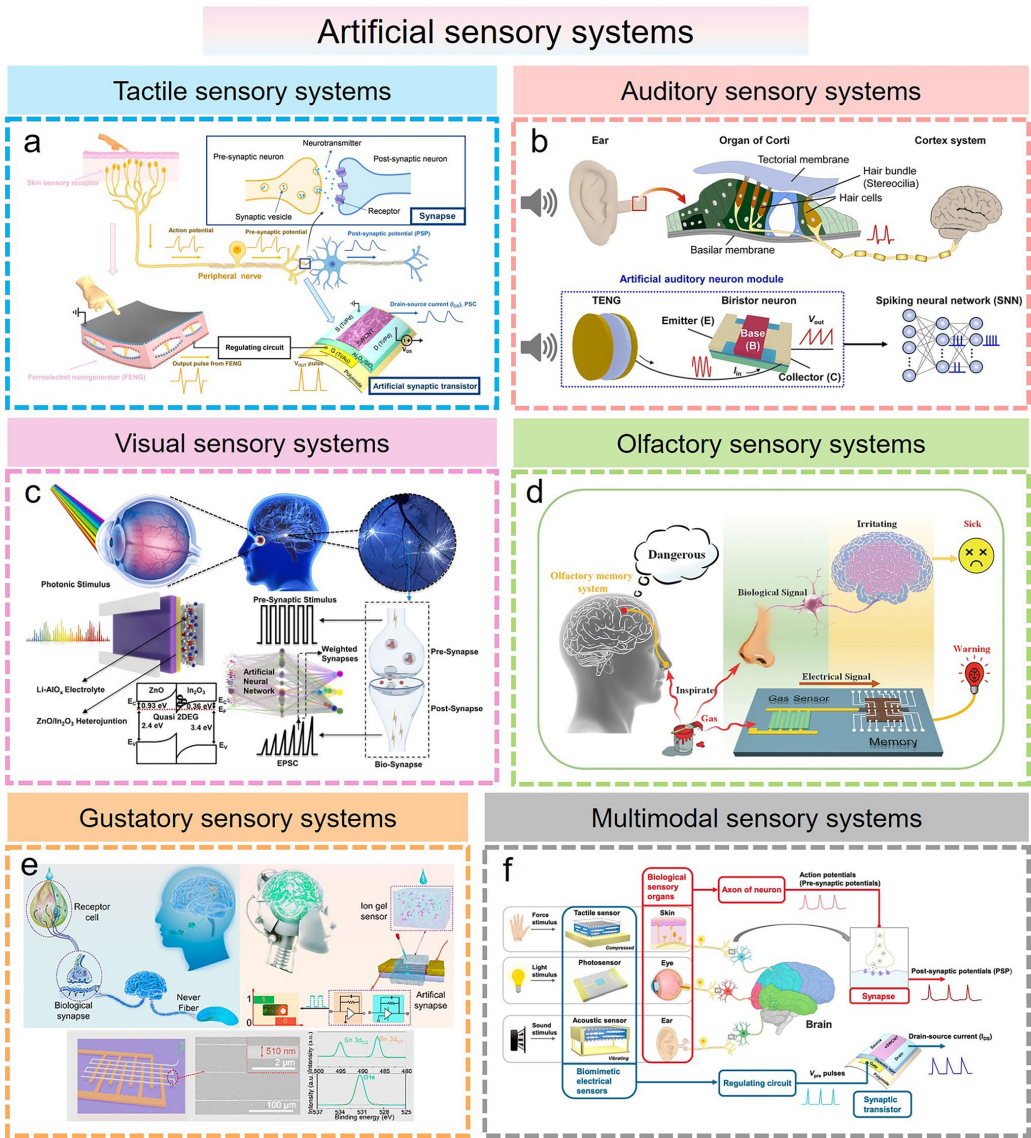
Various artificial sensory systems based on artificial synapse-driven smart flexible sensors. **a** Tactile sensory systems [31] Copyright (2020) American Chemical Society. **b** Auditory sensory systems [177] Copyright (2023) Elsevier. **c** Visual sensory systems [178] Copyright (2022) Elsevier. **d** Olfactory sensory systems [180] Copyright (2021) Wiley–VCH. **e** Gustatory sensory systems [181] Copyright (2023) American Chemical Society. **f** Multimodal sensory systems [179] Copyright (2021) American Chemical Society

In the human sensory organs, various types of receptors are important components of the human sensory system. These sensory receptors can detect and transform different stimulus signals from the surrounding environment, which in turn send interpretable sensory information to the brain for cognitive processing, thus achieving communication and socialization. This physiological process is accomplished through biological sensory organs combined with biological synapses. On this basis, five traditional sensory systems, including sight, hearing, smell, taste, and touch, are developed for humans to perceive the external world. With the rapid development of AI technology, intelligent flexible sensing systems that mimic bio-sensory organs are gradually needed to dynamically capture much of the physical information that describes the real world. Along with the explosive growth of information data, the signal processing and data analysis algorithms based on the traditional von Neumann architecture are no longer able to meet the increasing demand in terms of speed and efficiency, which triggers the burgeoning developments of brain-inspired synaptic devices. Artificial synapse is developed by mimicking the biological synaptic architecture, which can transmit and process sensory information in a manner similar to the biological neural networks responding to neural signals. Therefore, the incorporation of artificial synapses with flexible sensing elements can enable high-speed, efficient, low-energy parallel processing at multiple spatial and temporal scales, which will contribute to the design of intelligent flexible sensory systems, such as tactile, visual, auditory, olfactory, and gustatory sensory systems (Fig. 7 and Table 3).

**Table 3:** Summary of artificial sensory systems

Category	System architecture	Basic function	Application	References
Tactile sensory systems	Flexible ferroelectret nanogenerator, synaptic thin-film transistor	STP/LTP, STDP/SADP/SWDP	Neuroprosthetic control	[31]
	Pressure sensor clusters, ring oscillators, synaptic transistors	Artificial afferent nerve, synaptic reflex arc	Neurorobotics, neuroprosthetics	[182]
	Pressure sensor, threshold control unit, actuator	Artificial reflex arc	Soft robot, neuroprosthetics	[183]
Visual sensory systems	Metal-oxide heterojunction artificial synapses	STP/LTP, EPSC	Neuromorphic computing, visual sensory nervous	[178]
	Multifunctional sensors, flexible synaptic transistors	STM/LTM	Cyborg systems and neuroprosthetics	[179]
Auditory sensory systems	Triboelectric nanogenerator, bi-stable resistor	SNN, spiking output	Pitch classification, sound recognition	[177]
	triboelectric acoustic sensor, synaptic transistor	STP/LTP, PPF, SNDP/SDDP/SFDP/ SVDP	Neural prosthetics, bio-interface devices	[32]
Olfactory sensory systems	Gas sensors, memory unit	HRS/LRS	Artificial electronic nose	[180]
	Gas sensor, resistor electrochemical actuator	Sensing, memory and self-protection	Artificial nose, humanoid robot	[184]
Gustatory sensory systems	Ion-gel sensors, synaptic devices, execution unit	Salt-taste perception, excessive-intake warning	Taste health monitoring	[181]

*STM* short-term memory, *LTM* long-term memory, *PPF* paired-pulse facilitation, *HRS* high resistance states, *LRS* low-resistance states

The tactile perception system greatly facilitates human–environment interaction by receiving and processing external tactile information. Recently, several flexible electromechanical sensors have been proposed to achieve simple tactile perception in soft robotics and human–machine interaction. However, the large area of sensing signal processing will inevitably lead to high power consumption, heat generation, and delay. To solve these issues, artificial synapses based on memristors and transistors have been developed to mimic the parallel processing capabilities of the human brain [176]. Several studies have been conducted by combining different types of flexible electromechanical sensors with artificial synapses to build intelligent artificial sensory systems that can automatically perform data acquisition, processing, and analysis, and execute decisions and feedback actions based on learned knowledge. These artificial sensory systems consist of three main stages: sensing, signal processing, and perceptual decision-making. In general, the design and material selection of flexible sensors can mimic the soft and deformable features of human skin, allowing for a comfortable fit when in contact with the human body. In the sensing stage, flexible electromechanical sensors, as an important module of artificial tactile sensory systems, can perceive various tactile information (pressure, strain, and shape) by contacting objects and subsequently convert them into electrical signals. These acquired electrical signals are pre-processed and then transmitted to the brain-inspired synaptic device for further processing. This synaptic device is capable of processing perceptual information and adaptive learning, which can even be adjusted and optimized for various tactile environments and task requirements based on the pattern and frequency of the input signal. Specifically, the brain-inspired synaptic device can identify and extract key features in tactile information, such as object shape, hardness, and surface texture, by using pattern recognition and learning algorithms. Based on these features, the tactile sensory system can make appropriate decisions, such as object classification and texture evaluation. These decisions are further feedback to the device, endowing it with the ability to adapt and respond to external tactile stimuli. In recent research, a biomimicking sensory electronic skin system has been developed by integrating a flexible ferroelectret nanogenerator with a high-performance synaptic transistor (Fig. 7a) [31]. Several biological synaptic neurological functions based on the flexible synaptic transistor were successfully implemented, including temporal synaptic behaviors (STP/LTP), high-pass filtering characteristics, paired-pulse facilitation, and long-term learning and memory (STDP/SADP/SWDP). In this neurological electronic skin, flexible nanogenerators can serve as sensory mechanoreceptors, converting tactile inputs (both the amplitude and frequency of force) into pulsed electrical signals. These signals are then transmitted to the gates of synaptic transistors to render changes in their postsynaptic currents, mimicking synaptic weight modulation in biological synapses. In this manner, real human skin tactile perceptual behavior is successfully emulated through the biological synaptic transmission, processing, and memory of tactile signals. This bionic sensory electronic skin system can even be connected to an actuator unit for neuroprosthetic control. Similar to tactile sensory systems, several electromechanical sensors can detect sound waves of various frequencies and amplitudes, which can be combined with artificial synapses to create artificial auditory sensory systems (Fig. 7b) [177]. In this combination, flexible electronics serve as auditory receptors, converting sound waves into electrical signals. These signals are further transmitted to the synaptic device for processing, thus enabling the perception and understanding of auditory information.

Unlike tactile and auditory perception, the human visual system mainly relies on the perception of light signals to obtain visual information about the external environment and transmit it to the brain. To mimic this process, some researchers have developed artificial visual neuromorphic systems based on a combination of flexible optoelectronic sensors and artificial synapses, which provides a new direction for AI vision applications. In such a visual system, flexible optoelectronic sensors are responsible for sensing and collecting visual information, while synaptic devices are responsible for information processing and pattern recognition, working together to achieve perception and understanding of the visual world. Flexible optoelectronic sensors can perceive visual features such as light, color, and texture, and convert this information into electrical signals to artificial synapse modules. These artificial synaptic units can process and analyze the received visual information to extract advanced visual features as visual output, which are further used to develop several vision applications such as image recognition, scenario analysis, and motion tracking. Recent research on all-in-one artificial synapses has been proposed, which use metal-oxide ZnO/In_2_O_3_ heterojunction to integrate visual sensory and central nerve functions (Fig. 7c) [178]. Various synaptic plasticity functions such as long-term and short-term memory behaviors have been realized by modulating parameters such as the amplitude, width, frequency, and the number of electrical stimuli. This emulation of synaptic plasticity, along with the sustained synaptic weight state, enables the application of artificial synapses in neuromorphic computations, such as ANN-based image recognition of the Covid-19 chest (> 85%). In addition, such artificial synapses are capable of responding to optical and UV stimuli, thus mimicking biological visual sensory functions. This all-in-one artificial synapse combining neuromorphic computing and visual perception functions demonstrates a broad promise in future AI systems. Notably, the fusion of flexible multifunctional sensors with artificial synapses can even achieve multimodal artificial sensory-memory systems (Fig. 7f) [179]. The system is made up of multifunctional sensors for tactile, auditory, and visual inputs and flexible synaptic transistors with signal processing and memory behavior. In such a system, the physical stimuli are converted into electrical impulses containing various information, which are then transmitted to an artificial nervous system based on flexible CNT synaptic transistors for processing and storage. On this basis, biological receptor-like perception and synaptic memory behavior were successfully achieved. Several scenarios such as the “multistore memory” model (Atkinson–Shiffrin memory model) and the classical conditioned reflex experiment (Pavlov’s dog experiment) were also demonstrated.

Olfactory and gustatory perceptions endow organisms with sensitivity and adaptability to the external environment by perceiving and discriminating odors and chemical substances, which are extremely important for survival, evolution, and self-protection. Nowadays, the combination of flexible chemical sensors and artificial synapses opens a new possibility for the development of artificial olfactory and gustatory sensory systems, such as an electronic nose for smell and an electronic tongue for taste (Fig. 7d, e). In these systems, flexible chemical sensors sensitive to specific gas or chemicals enable olfactory and gustatory information acquisition, while artificial synapses are responsible for signal processing to achieve understanding and analysis of olfactory and gustatory information. Specifically, functional sensing materials can react chemically with a target gas or chemical to generate a specific electrical signal or resistance change. Then, the brain-inspired synaptic device utilizes neural networks and algorithms to recognize and analyze these signals and output the corresponding feedback results based on the learning and memory functions. For example, Choi et al*.* reported an artificial olfactory memory system to mimic human olfactory memory by combining gas sensors with resistive switching memory (Fig. 7d) [180]. This gas sensor with high sensitivity to VOC molecules can convert the VOC signal into an electrical signal to trigger the memory device to provide smell sensation retention and perform gas information identification tasks. Moreover, a smart robot equipped with this artificial olfactory memory system was utilized to demonstrate the visualization of gas-sensing. In short, the collaboration between flexible electronics and artificial synapses will further advance the development of artificial perception systems and enable more intelligent and personalized services and experiences for humans.

### Fusion of Flexible Sensors with Machine Learning and Artificial Synapse

More importantly, in addition to simple machine learning-driven or artificial synapse-driven smart flexible sensors, the fusion of flexible sensors with machine learning and artificial synapses will lead to more sophisticated intelligent wearable applications with the integrated functions of recognition, sensing, memory, computing, and feedback. However, the present research work on the fusion of the three is relatively limited. For example, in several advanced artificial auditory systems, artificial synapse modules that integrate a series of neural networks and machine learning algorithms can perform the recognition, analysis, and processing of sound signals collected by various auditory sensors. These artificial synapses are able to extract auditory features from massive perceptual data and perform tasks such as sound localization and recognition, speech analysis, and environmental noise suppression. As a demonstration, Choi et al*.* demonstrated an artificial auditory neuron module that integrates a bi-stable resistor (biristor) and a triboelectric nanogenerator (TENG) [177]. In this system, the TENG works as a power harvester and sound sensor, while the neuronal resistor takes on the role of a spiking neuron to implement data processing by combining machine learning algorithms. The TENG can perceive acoustic pressure to generate electrical signals as inputs to the collectors of biristor neurons, yielding a spiking output voltage for the spiking neural network (SNN). This SNN-based self-aware artificial auditory system was further applied for instrument pitch classification in piano or sound recognition in cello and violin. Similar work was also exhibited by Xu’s group in 2022, who developed a stretchable neuromorphic transistor with the capabilities of visual and tactile information perception and neuromorphic processing [185]. Therefore, several biological synaptic functions are successfully simulated, including SFDP, SNDP, EPSC, and PPF. This artificial synapse with strain sensing function can be further used as a tactile afferent nerve and combined with a machine learning model of ANN to realize gesture recognition function with an accuracy of up to 96.3%. Although these studies are in their infancy and achieve less neural function than biological systems, it points a key direction to the more complex fusion of flexible sensors with machine learning and artificial synapses.

## Applications Based on AI-Driven Smart Flexible Sensing Systems

### More Intelligent Monitoring for Human Activities

**Fig. 8:**
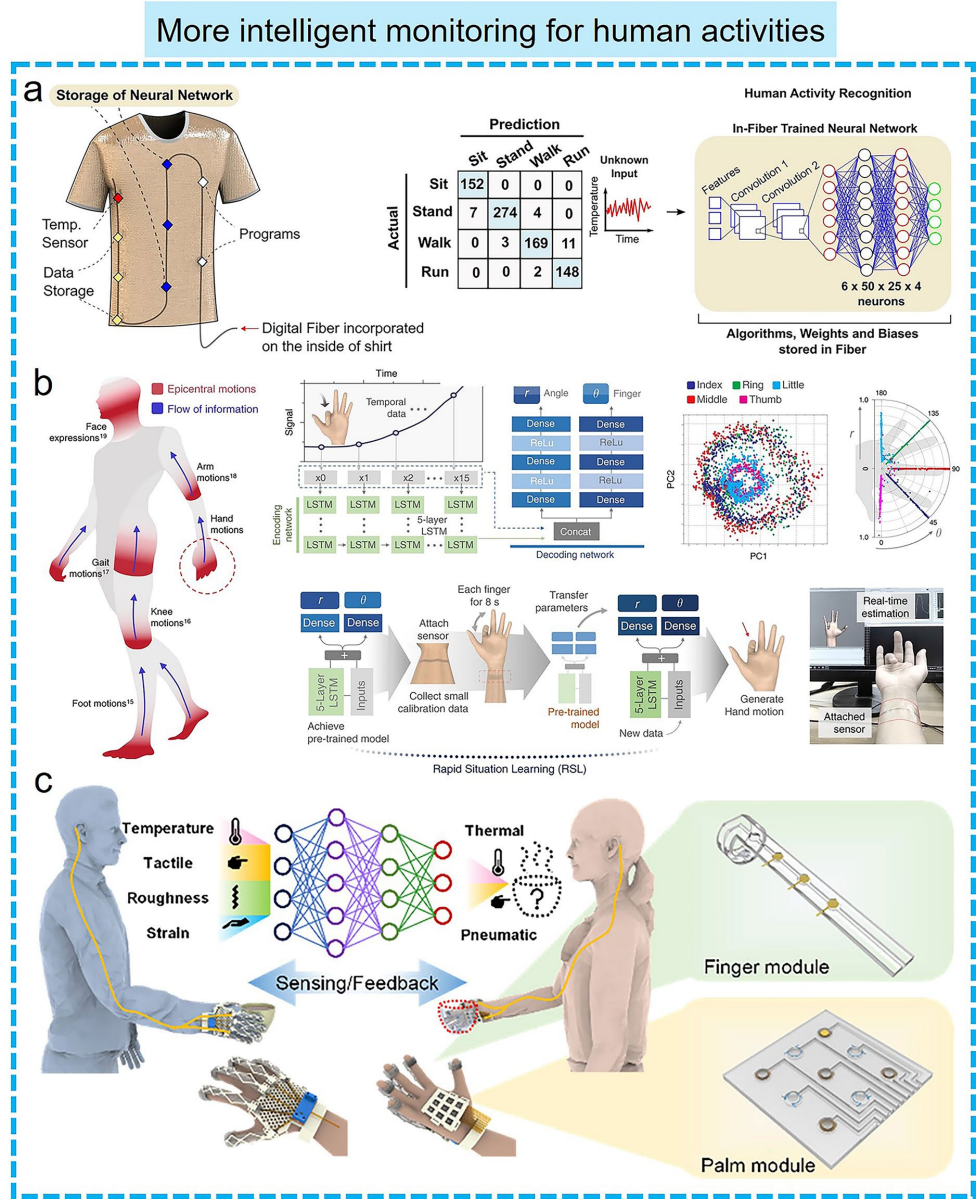
AI-driven smart flexible sensing systems for more intelligent monitoring of human activities. **a** Fabric with incorporated neural network capabilities for inferring wearer activity [187] Copyright (2021) The Authors. **b** A deep-learned skin-like sensor system decoding the human hand movements from detected skin deformation [188] Copyright (2020) The Authors. **c** Multimodal sensing and augmented haptic feedback soft modular glove for human–machine interaction [44] Copyright (2022) American Chemical Society

In the era of AI and big data, various smart sensing systems with intelligent perception, autonomous decision-making, and self-adaptive capabilities are developed based on flexible electronics, which play an irreplaceable role in the field of human activities and services. These devices can provide more natural and intuitive interaction, personalized and customized services, achieving intelligent environmental interaction, and enhanced virtual reality experience. For instance, flexible tactile sensors can be directly attached to the skin surface or integrated into clothing to detect human motion, thus enabling posture adjustment and human–machine interaction. Matusik et al*.* reported a tactile learning platform based on coaxial piezoresistive fibres that combine t-distributed stochastic neighborhood embedding (t-SNE) and CNN algorithms to classify human sitting, movements, and other human–environment interactions [186]. Using their full-size sensing vest, various postures such as sitting, standing, and reclining can be distinguished based on the characteristic pressure distribution of various actions. Similarly, Fink’s group developed a scalable flexible fiber that contains hundreds of scattered temperature sensors and storage devices [187]. This digital fiber can be incorporated into shirts to collect and store multiple days of body temperature data and infer the wearer’s activity (sitting, standing, walking, and running) in real time with an accuracy rate of up to 96% by using a trained neural network model (Fig. 8a). Moreover, a novel ultra-sensitive skin sensor has also been proposed to decode the epicentral human motions (Fig. 8b) [188]. This sensor can measure small skin deformation signals away from the joints and combine them with DNN algorithms to monitor the motion of the corresponding body part. As proof, when attached to the wrist or pelvis, the sensor is capable of extracting signals corresponding to multiple finger movements or generating dynamic gait movements. Based on the capability of human motion detection and posture recognition, these wearable sensors are further used to develop sign language translation functions aimed at helping disabled people overcome communication barriers. Zhu et al. combined stretchable yarn-based sensor arrays with printed circuit boards to build wearable sign-to-speech translation systems [108]. This system can achieve accurate translation from hand gestures to speech based on the assistance of machine learning algorithms and standard American Sign Language (ASL).

Additionally, in healthcare and disease diagnosis, wearable sensors are urgently needed for monitoring various physiological signals, such as blood pressure, blood oxygen, pulse, breathing, EMG, and ECG. Wu et al*.* [189] designed a triode-mimicking flexible graphene pressure sensor with a broad working range and an ultrahigh sensitivity. This device with a mechanical triode-like signal amplification characteristic can be used to accurately detect a variety of human movements and subtle physiological signals, including plantar pressure/gait, breathing, and pulse detection. On this basis, AI-based integrated gait monitoring and arterial blood pressure detection systems are developed. Rogers et al*.* have implemented mechanical acoustic sensing of body movements and physiological processes by designing a wearable wireless device and combining it with a hidden Markov model algorithm for data analysis and processing [126]. This soft wireless device is attached to the suprasternal notch to collect multimodal information in connection with various physiological processes, including heart rate, speaking time, number of swallows, and energy expenditure. Subsequently, these raw physiological data are analyzed and classified by machine learning algorithms using the time and frequency domains. In addition, human–machine interaction based on wearable sensor systems is gradually becoming an important component of daily life, such as smart homes, virtual reality, games and entertainment, and public transport. Recently, a modular soft glove with augmented haptic feedback and multimodal sensing functions has been designed by Lee's group (Fig. 8c) [44]. Combined with machine learning algorithms, this smart glove not only detects dexterous hand movements in real time, but also enables accurate object recognition and enhanced feedback, significantly enhancing the perception and communication of more comprehensive information. As a demonstration, the glove was further used for bi-directional and multimodal communication between humans, robots, and virtual worlds. Similarly, Zi et al*.* reported a porous-structure-promoted self-powered tactile sensor to construct a programmable optoelectronic dual-mode human–machine interaction system that can remotely control smart vehicles and operate computer games by recognizing finger-touch trajectories [190]. In a word, the wave of AI and information technology will certainly inspire more sophisticated and smarter flexible electronic design, driving revolutionary progress in healthcare, education, transportation, human life, and many other fields.

### More Humanoid Feeling by Artificial Sensory Organs

**Fig. 9:**
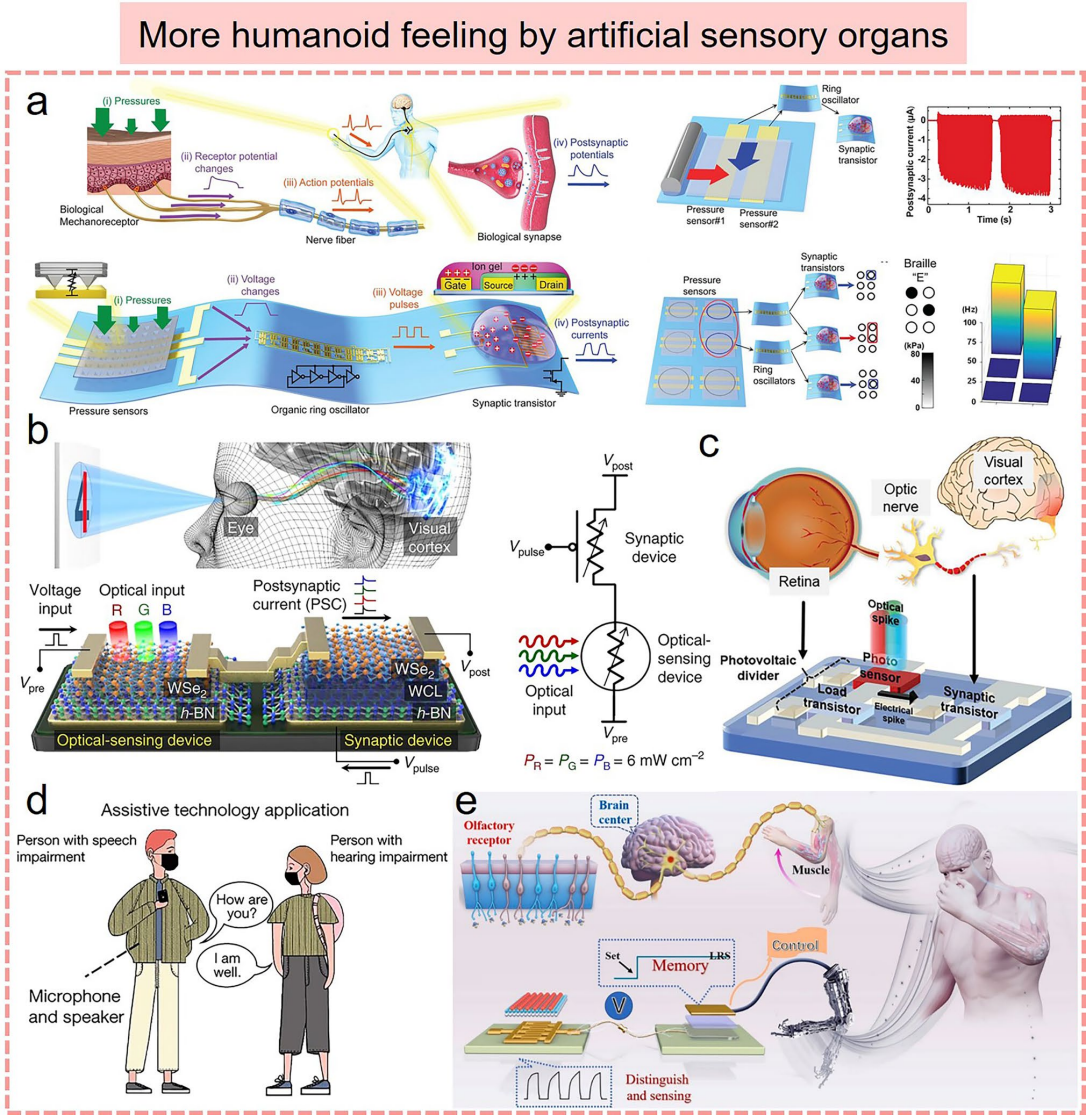
AI-driven smart flexible sensing systems for more humanoid feeling by artificial sensory organs. **a** A bioinspired flexible organic artificial sensory nerve for movement recognition and braille reading [182] Copyright (2018) The Authors. **b** An artificial optic-neural synapse system for pattern recognition [193] Copyright (2018) The Authors. **c** An artificial optoelectronic neuromorphic circuit array for mimicking environment-adaptable artificial visual sensory system [195] Copyright (2019) Wiley–VCH. **d** A planar acoustic fabric for bidirectional communications [198] Copyright (2022) The Authors. **e** A complete artificial olfactory system for odor perception, recognition, memory, and self-protection [184] Copyright (2021) Elsevier

Nowadays, five artificial sensory systems with more humanoid feelings developed by artificial synapse-driven smart flexible sensors are becoming an indispensable element for human life, as illustrated in Fig. 9. Among biological sensory systems, tactile perception relies on various sensory receptors on the skin surface to interact with the external environment. Recently, artificial tactile systems that mimic human skin perception have been developed for various fields such as smart wear, medical monitoring, prosthetics, intelligent robotics, and artificial tactile sensory memory [11, 94]. As proof, Bao’s group developed a skin-inspired digital tactile system by combing a pyramid-shaped tactile sensor with an organic oscillator, which can convert pressure into a digital frequency signal for direct output [191]. These devices can be further integrated into a wearable glove to exhibit the variation of the voltage frequency with the applied pressure in the wearable system. Similar work was also demonstrated by Lee’s group in 2018 (Fig. 9a) [182]. They reported a flexible organic artificial sensory nerve by a combination of pressure sensor clusters, ring oscillators, and synaptic transistors. This biomimetic nerve structure can distinguish braille characters based on simultaneous pressure inputs and even be used to construct hybrid bioelectronic reflex arcs in neurorobotics and neuroprosthetics. In addition, the design of the multimodal flexible tactile sensory system laid the foundation for achieving intelligent robot interaction. Wang’s group fabricated a skin-inspired flexible multifunctional sensor array based on the stacked and distributed layouts for constructing intelligent prostheses, demonstrating its implementation for temperature estimation and spatial pressure mapping [192]. For the development of artificial tactile sensory memory, researchers prefer to develop complete information acquisition, memory, and feedback execution systems to design more advanced intelligent robotic systems. Chen et al*.* developed an artificial somatic reflex system to mimic the reflex arc function of humans and higher animals [183]. Besides the pressure sensor and threshold control unit, this system includes an electrochemical actuator based on the multi-wall CNT for feedback of external pressure stimuli. This artificial reflex arc is further integrated into a 3D-printed robot to emulate infant grasp reflexes, demonstrating their promise in the development of neuroprosthetics and intelligent soft robots. In short, these artificial tactile sensory systems that simulate perception and memory may inspire more integrated and connected wearable devices in the future.

In visual perception, mimicking human light perception behavior based on biological synaptic function is essential for the development of artificial vision systems. Seo et al*.* [193] reported an artificial optic-neural synapse system to successfully mimic several synaptic functions by regulating the light conditions, such as long-term potentiation/depression and STDP (Fig. 9b). This synaptic system is also used for mixed-color and color pattern recognition in the human visual system. Currently, artificial vision systems based on different types of optoelectronic sensors and synaptic devices have been advanced in many fields, such as retinal bionic chips, flexible optoelectronic skins, artificial electronic eyes, and imaging sensors. Liu’s group integrated the ferroelectric/electrochemical modulated organic synaptic device with the light-sensitive electronic element to construct a light-triggered organic neuromorphic device (LOND) as a conceptual demonstration for artificial visual sensory systems [194]. The LOND can mimic retinal functionalities by converting photostimuli signals with various intensities, wavelengths, and frequencies into different types of synaptic signals. With the rapid development of artificial vision sensory systems, a biomimetic visual adaptation that can automatically adjust retinal sensitivity is proposed to construct advanced image-sensing systems with more accurate recognition and a wide detection range. Park’s group reported an artificial neuromorphic circuit array to mimic the light-adaptable function of the biological retina, exhibiting the environment-adaptable artificial visual sensory system (Fig. 9c) [195]. This artificial visual sensory system with photopic and scotopic adaptation behavior can enhance the accuracy and efficiency of the image recognition process. Furthermore, flexible photoelectronic skin based on artificial vision sensors is widely used in healthcare and medical diagnosis. Koo et al*.* [196] designed a wearable cardiac monitor by integrating the p-MOS CNT signal amplifier with the color-tunable OLED, which can continuously detect ECG signals based on the OLED corresponding color change from dark red to pale red, white, sky blue, and deep blue.

The auditory sensory system allows creatures to acquire the external environment information by perceiving sound waves of different frequencies and amplitudes. Nowadays, researchers are working to develop a lightweight, stable, and low-power artificial auditory sensory system to help hearing-impaired people communicate with each other. For example, intelligent robots integrated with artificial auditory systems can generate high-complex communication capabilities by locating and tracking sounds, such as simultaneous sound source separation and speech recognition [197]. In addition, several other scenes were also demonstrated. Fink et al*.* reported a planar acoustic fabric that can serve as a sensitive audible microphone (Fig. 9d) [198]. This acoustic fabric can monitor the wearer’s breathing in real time and even be used in maternity wear to monitor the heart rate of the fetus. Lee’s group also reported an artificial auditory sensory nerve by integrating the triboelectric acoustic sensor with the ion gel-gated organic synaptic transistor, enabling several sound wave-induced synaptic functions (STP/LTP and EPSC) [32]. Furthermore, Wang et al*.* [199] successfully performed sound localization utilizing the STP behavior of HfO_x_-based memristors. The sound source azimuth was accurately identified for the interaural time difference simulation by evaluating the internal potential difference between two postsynaptic neurons.

Compared with the other three perception systems, research works on artificial olfactory and gustatory sensory systems are relatively simple. Recently, several flexible chemical sensors have been used to design artificial olfactory and taste perception systems for food quality/safety assessment, pharmaceutical analysis, disease diagnosis, environmental monitoring, and bioelectronic nose [200]. For example, Liu’s group developed a conformable and flexible artificial organ-damage memory system to simulate the process of inhalation and cumulative organ damage in humans during hazardous gas (NO_2_) leakage, which was implemented using an organic field effect transistor to combine gas sensing detection with information storage functions [201]. Similarly, Shen et al*.* also designed a complete artificial olfactory system to perform odor perception, recognition, memory, and protection action behavior by combining a flexible gas sensor, a memory resistor, and an artificial muscle actuator, as shown in Fig. 9e [184]. As a demonstration, this system not only senses, recognizes, and real-time memorizes NH_3_, but also mimics self-protective actions that induce muscle movements. Moreover, Xu et al*.* reported an artificial neuromorphic gustatory system that can perform taste perception, and information processing functions, and make immediate responses and warning against the highly concentrated salt solution [181]. Although the current exploration of artificial olfactory and gustatory sensory systems is still in its infancy, it offers a promising strategy for mimicking and restoring biological smell and taste.

In brief, with the continuous progress and innovation of flexible electronics, artificial sensory systems based on the mimicking of human sensory organs will become more intelligent, flexible, and integrated, advancing science and technology society progress.

### More Autonomous Action of Soft and Humanoid Robots

**Fig. 10:**
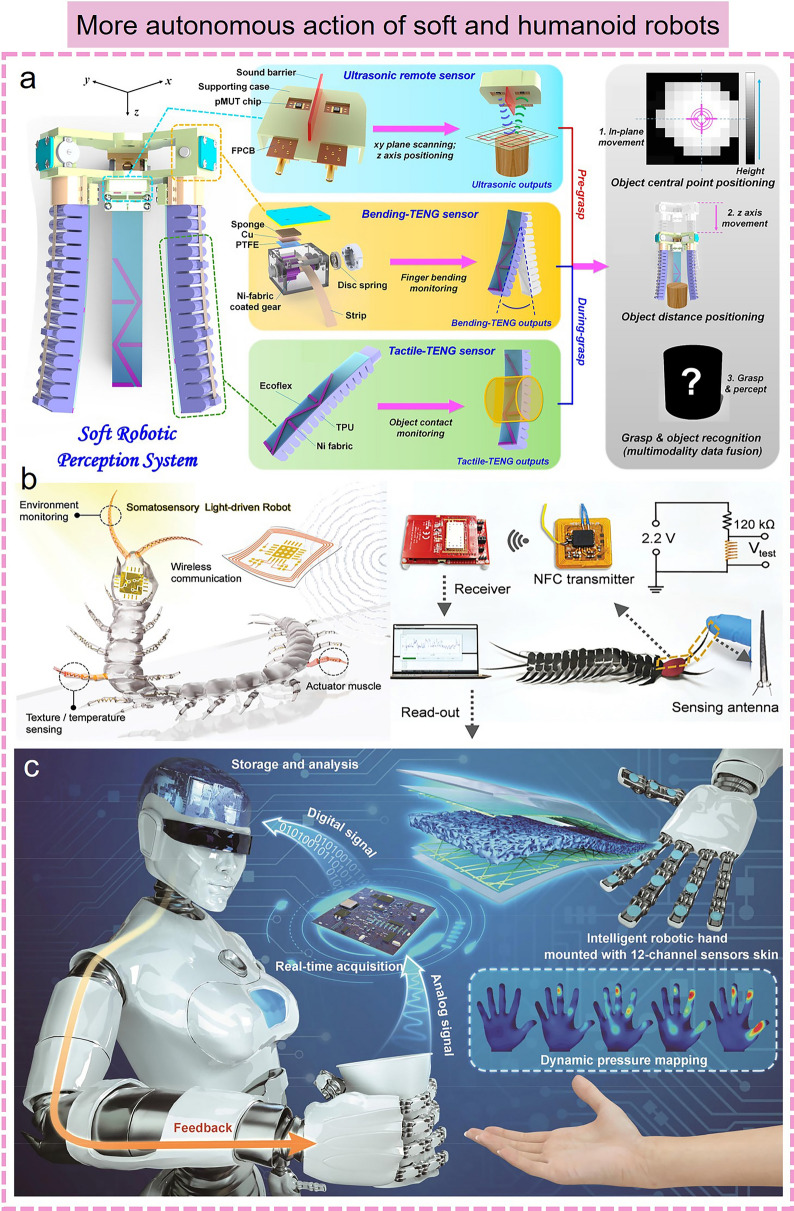
AI-driven smart flexible sensing systems for more autonomous action of soft and humanoid robots. **a** A soft robotic perception system for remote object positioning and multimodal sensory intelligence [202] Copyright (2023) American Chemical Society. **b** An SLiR-based untethered centipede with integrated perception and motion capabilities for directional movement, multisensory to wireless human–environment interaction [41] Copyright (2020) Wiley–VCH. **c** The humanoid robot integrated with an intelligent tactile system and AI technology for dynamic pressure sensing, data storage and analysis, and motion feedback [209] Copyright (2023) Elsevier

Soft robots with great flexibility and variability allow comfortable physical contact for operation and thus are widely used in many dynamic, non-planar scenarios, such as gripping, manufacturing, manipulation, locomotion, and human–machine interaction. The integration of artificial sensory systems and soft robots can endow robots with human-like perception and interaction functions, which enhances the adaptability, flexibility, and safety of robots, enabling them to better understand and adapt to the surrounding environment. For example, soft robots based on tactile sensory systems can flexibly manipulate objects and perform high-precision assembly tasks. In order to achieve convenient autopositioning and a multimodal cognition capability of soft robots, Lee et al*.* developed a soft robotic perception system by integrating ultrasonic sensors with flexible tactile sensors on a robotic manipulator (Fig. 10a) [202]. The manipulator is moved to the right position for object grasping based on the object shape, height, and distance information feedback from the ultrasonic sensor. This process also includes the acquisition of multimodal sensory information such as object shape, size, hardness, and material by flexible triboelectric and tactile sensors. Combined with the training and optimization of DL networks for data analysis, the perception system can achieve 100% accuracy in object classification. This multimodal sensing and positioning system based on soft robots demonstrates great potential in unmanned stores, automatic sorting, smart manufacturing, and healthcare assistance. Similar work for object recognition and perception was also demonstrated by Zhu’s group and Duan’s group. Zhu’s group reported a robotic hand with an integrated quadruple tactile sensor that can simultaneously and independently perceive multiple stimuli such as contact pressure, material thermal conductivity, object, and ambient temperature to accurately recognize various objects [203]. Further incorporating machine learning, this intelligent robotic hand demonstrated 94% classification accuracy in a garbage sorting task. Duan et al*.* developed a multifunctional soft finger by embedding a built-in pressure and temperature tactile sensor [204]. With high sensitivity and low cross-sensitive interference, this smart soft finger can identify four metals and 13 other materials.

Soft robots with closed-loop control systems can achieve self-manipulation and movement, which is extremely important for safety protection, accident rescue, and information acquisition in complex environments. Wang et al*.* [41] reported a somatosensory light-driven robot (SLiR) by using a smart thin-film composite with tightly integrated actuation and multiple sensing, which can simultaneously and independently perceive its body temperature and actuated deformation state by using a photoactuator transducer. This SLiR with coordinated actuation, proprioception, and communication is suitable for a variety of complex scenarios, including feedback on walking gait and evaluation of terrain texture. A SLiR-based anthropomorphic hand is designed to provide similar somatosensory reception, such as specific finger movements, hot and cold sensations, and soft and hard perceptions. Furthermore, SLiR-based centipede can perform different, localized bodily functions ranging from directional movement and multisensory to wireless human–environment interaction (Fig. 10b). Zhou et al*.* [205] also developed a 3D-printed multifunctional wearable sensor to endow a snake-like soft robot with the ability to distinguish tensile and bending deformation. As an example, this snake-like robot can provide feedback on the endoscope position by measuring finger curvature, exhibiting good practicality in posture detection. Moreover, soft robots based on various artificial perception systems have demonstrated undeniable value in the medical fields, including drug delivery, minimally invasive surgery, disease diagnosis, and rehabilitation training. In a recent study, Stoyanov’s group designed a fluid-driven soft robot with an integrated visual perception system for positioning and tracking, which can actively manipulate the inserted needle to reduce the risk of intratympanic injections [206]. By detecting the desired injection point to avoid unnecessary movement, the integrated visual perception system further reduces the procedure risk by controlling the image guidance function of the robot. Several types of artificial electronic eyes based on visual sensory systems have also been developed to emulate the visual function of the human eye, which is essential for robotics and visual prostheses. Gu et al*.* [207] reported a biomimetic eye based on an artificial vision system. Similar to the human eye structure, this biomimetic eye possesses a hemispherical retina consisting of high-density nanowire arrays that mimic the photoreceptors in the retina of humans, thus enabling high-resolution image sensing. Yan et al*.* reported a multifunctional robotic vision system that is able to recognize, memorize, and initiate self-protection functions by integrating two memristor units with an electrochemical actuator [208]. When the human eyes are damaged by bright light, it can simulate the self-protective action of closing the eyes.

In addition to several soft robots such as prosthetics and manipulators, flexible electronics combined with AI technology can ensure that humanoid robots with more complex environmental adaptability and interaction capabilities are closer to the level of human perception and intelligence, inspiring more possibilities for future intelligent robot applications. For instance, artificial tactility that mimics human tactile sensory functions has been realized through the combination of humanoid robots and AI. Bao et al*.* [209] designed an intelligent tactile system with a closed-loop control characteristic to endow humanoid robots with human-like tactile perception, which is composed of a high-performance tactile sensors array, a real-time information acquisition/interpretation chip, and a feedback control module (Fig. 10c). By combining AI technology with this intelligent tactile system, humanoid robots can easily achieve dynamic pressure sensing, data storage and analysis, and real-time motion feedback, exhibiting great promise in space manipulators, home entertainment, and smart home. Similar work to achieve stable tactile perception and mechanical endurance in humanoid robots was accomplished by Ling's group [210]. They developed an ionotronic skin to endow humanoid robots with smart object recognition by finger touching or tapping. This ionotronic skin can accurately sense triboelectric signals. Further by combining the trained recurrent neural network model, the humanoid robot can recognize spherical objects of various materials with a success rate of 97.2% and transport them to the specified location. Therefore, this intelligent humanoid robot can be used for automated sorting and assembly in smart factories. Moreover, Massari et al*.* [211] developed a large-area sensitive bionic skin embedded with grating sensors and mechanoreceptors, which can cover the whole body of a humanoid robot with modular patches and be combined with a CNN algorithm to predict the magnitude of the contact force. With the continuous development of flexible electronics, various complex flexible sensing systems will play a more important role in soft robots, humanoid robots, and human–machine interfaces in the future.

## Conclusion and Outlook

The past few years have witnessed significant advancements in AI-incorporated smart flexible sensor systems. In this review, we summarized recent developments regarding this topic.

With the advantage of highly efficient information processing and high-quality feature recognition, machine learning is well-suited for large-scale sensing data analysis, interpretation, and mode determination. Besides, the capability of machine learning to decouple multimodal/types of information contributes to a more accurate understanding of comprehensive sensing data obtained in complex practical environments. On the other hand, mimicking the working mechanism of the human brain, artificial synapses feature low power consumption, high parallelism, and real-time processing capabilities, which are highly promising as next-generation computing devices that are beyond traditional von Neumann architectures. This emerging neuromorphic framework inspires the design of various intelligent artificial sensory systems. It can be witnessed that the deep incorporation of these two AI technologies will trigger a more significant evolvement of traditional flexible sensors into smarter flexible sensing systems, which not only collects information from the external environment, but also intelligently analyzes and interprets the data to achieve “self-perception” of the environment. These new enabling features are essential for broad applications such as intelligent soft robotics, electronic glove/skins, human–machine interface, etc.

Despite remarkable advancements achieved, the development of AI-driven smart flexible sensor systems still presents substantial challenges. For the better incorporation of machine learning, (1) it should be noted that the acquisition of massive experimental data with high consistency is still time-consuming and challenging for many flexible electronic applications. More advanced algorithms should be developed to alleviate the requirements of data acquisition for model training. (2) Besides, flexible electronics are often operated in diverse dynamic or even harsh applications scenarios, which in turn gradually changes the physical/chemical properties of the constituted materials of the devices. The adaptability of the as-trained models to flexible electronics with changing properties remains a question. (3) Another doubt aroused by this issue is that: due to unavoidable variation in material properties and fabrication process, can a well-trained machine learning model on one flexible device be smoothly transferred/applied on another one of the same kind? (4) Currently, the computational power of flexible electronics is inferior to common computer hardware, while the model training of machine learning requires massive computational capacity. It remains a long way to go to realize real-time model updating on the deployed flexible devices. As for the fusion of artificial synapses for smart flexible sensing, (1) although many synaptic and flexible devices have been developed in recent years, the flexibility of such devices should be continuously improved, especially the synaptic behavior and performance durability in harsh scenarios such as twisting and stretching. (2) Synaptic-based intelligent sensory system relies on integration of the flexible sensors with synaptic components, where the interfacing and communication between them made of diverse or even distinct materials remains a challenge. (3) From a system-design level, the construction of intelligent flexible sensing requires more cooperative developments of flexible sensors, machine learning algorithms, and artificial synapses. In conclusion, with the increasingly deeper engagement between AI technology and flexible sensing, we are approaching a new era of smart society.
